# Supplementary material for: Edge and texture aware image denoising using median noise residue U-net with hand-crafted features
Source: PeerJ Comput Sci. 2025 Jan 16;11:e2449. doi: 10.7717/peerj-cs.2449 (PMC11784896; doi:10.7717/peerj-cs.2449)
Supplement: Supplemental Information 1 — Training and testing dataset [file peerj-cs-11-2449-s001.docx]

**DATA AVAILABILITY STATEMENT**

For Training: **BSD dataset-** <https://www2.eecs.berkeley.edu/Research/Projects/CS/vision/bsds/>

For Testing: SET12, CBSD68, KODAK24, and McMaster datasets were used. The testing dataset was obtained from Kaggle and uploaded to Figshare. A link to the dataset is provided for easy access. <https://doi.org/10.6084/m9.figshare.26827765.v1>
