# Supplementary figures and images for: Edge and texture aware image denoising using median noise residue U-net with hand-crafted features (part 2 of 2)
Source: PeerJ Comput Sci. 2025 Jan 16;11:e2449. doi: 10.7717/peerj-cs.2449 (PMC11784896; doi:10.7717/peerj-cs.2449)

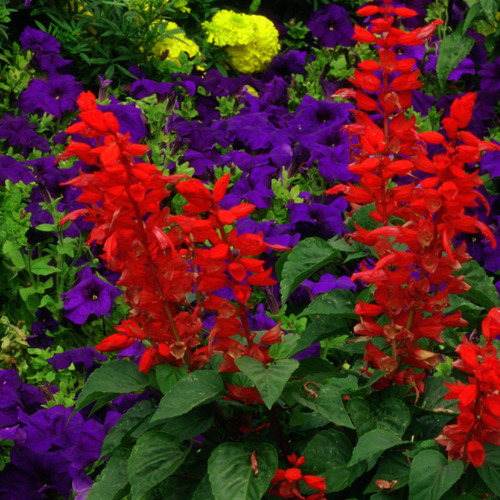

Supplement: Supplemental Information 2 — For Training purposes- Berkeley Segmentation dataset300 was used named soniya-mask. CBSD68, Set12, McMaster, and Kodak24 were used for testing purposes. [file peerj-cs-11-2449-s002.zip › overall database/Mcmaster/17.jpg]

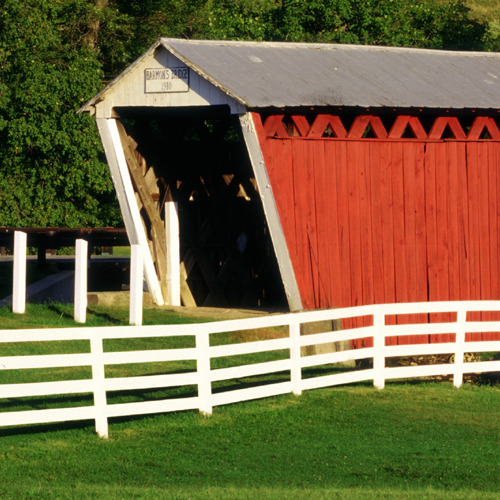

Supplement: Supplemental Information 2 — For Training purposes- Berkeley Segmentation dataset300 was used named soniya-mask. CBSD68, Set12, McMaster, and Kodak24 were used for testing purposes. [file peerj-cs-11-2449-s002.zip › overall database/Mcmaster/18.jpg]

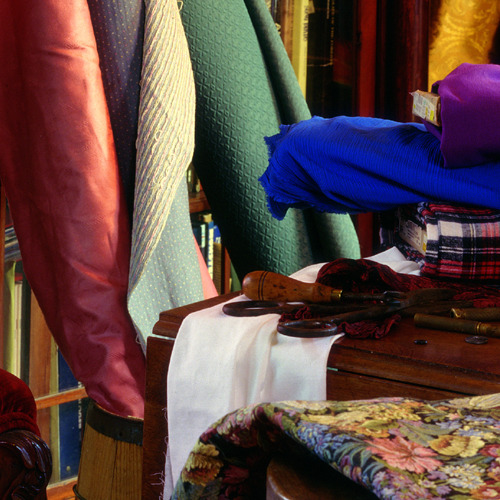

Supplement: Supplemental Information 2 — For Training purposes- Berkeley Segmentation dataset300 was used named soniya-mask. CBSD68, Set12, McMaster, and Kodak24 were used for testing purposes. [file peerj-cs-11-2449-s002.zip › overall database/Mcmaster/2.jpg]

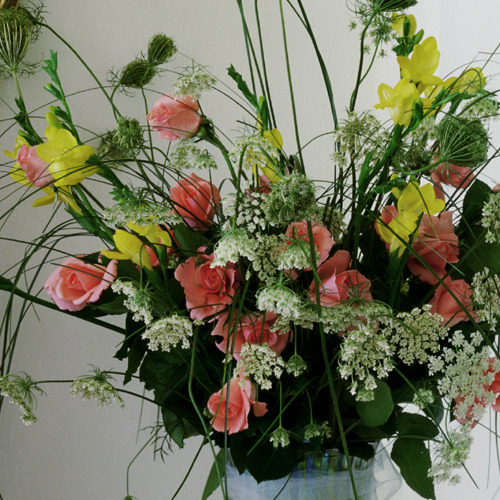

Supplement: Supplemental Information 2 — For Training purposes- Berkeley Segmentation dataset300 was used named soniya-mask. CBSD68, Set12, McMaster, and Kodak24 were used for testing purposes. [file peerj-cs-11-2449-s002.zip › overall database/Mcmaster/3.jpg]

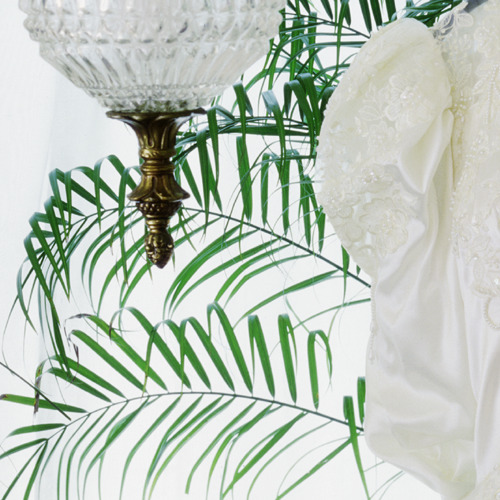

Supplement: Supplemental Information 2 — For Training purposes- Berkeley Segmentation dataset300 was used named soniya-mask. CBSD68, Set12, McMaster, and Kodak24 were used for testing purposes. [file peerj-cs-11-2449-s002.zip › overall database/Mcmaster/4.jpg]

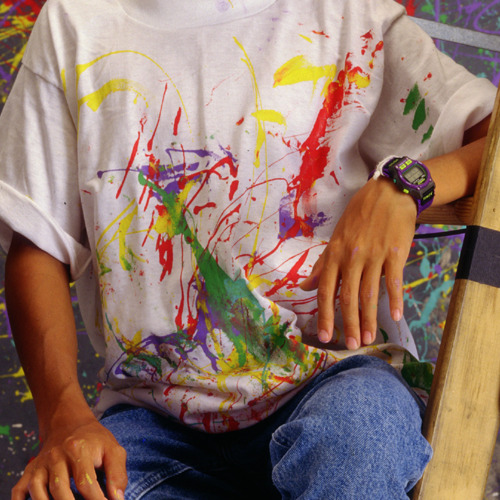

Supplement: Supplemental Information 2 — For Training purposes- Berkeley Segmentation dataset300 was used named soniya-mask. CBSD68, Set12, McMaster, and Kodak24 were used for testing purposes. [file peerj-cs-11-2449-s002.zip › overall database/Mcmaster/5.jpg]

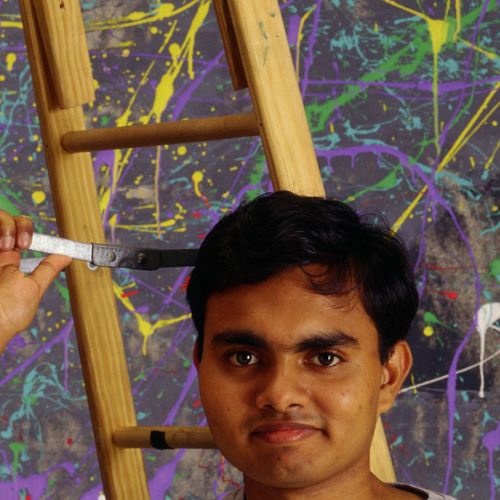

Supplement: Supplemental Information 2 — For Training purposes- Berkeley Segmentation dataset300 was used named soniya-mask. CBSD68, Set12, McMaster, and Kodak24 were used for testing purposes. [file peerj-cs-11-2449-s002.zip › overall database/Mcmaster/6.jpg]

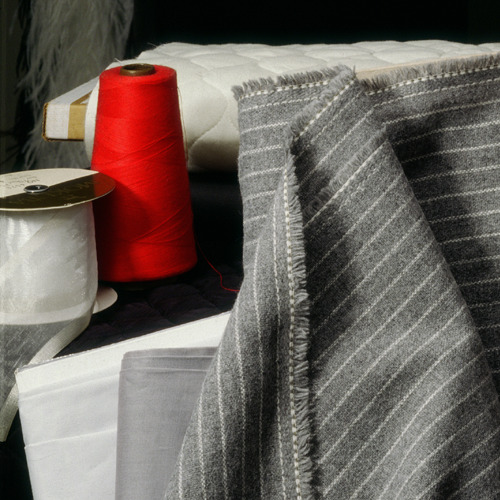

Supplement: Supplemental Information 2 — For Training purposes- Berkeley Segmentation dataset300 was used named soniya-mask. CBSD68, Set12, McMaster, and Kodak24 were used for testing purposes. [file peerj-cs-11-2449-s002.zip › overall database/Mcmaster/7.jpg]

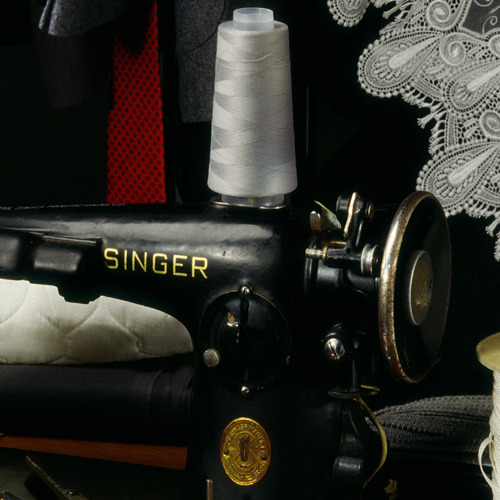

Supplement: Supplemental Information 2 — For Training purposes- Berkeley Segmentation dataset300 was used named soniya-mask. CBSD68, Set12, McMaster, and Kodak24 were used for testing purposes. [file peerj-cs-11-2449-s002.zip › overall database/Mcmaster/8.jpg]

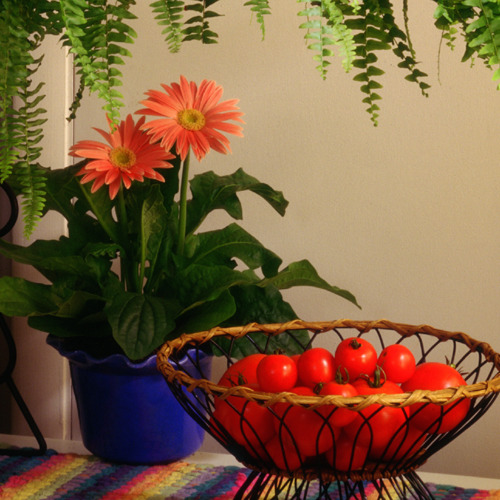

Supplement: Supplemental Information 2 — For Training purposes- Berkeley Segmentation dataset300 was used named soniya-mask. CBSD68, Set12, McMaster, and Kodak24 were used for testing purposes. [file peerj-cs-11-2449-s002.zip › overall database/Mcmaster/9.jpg]

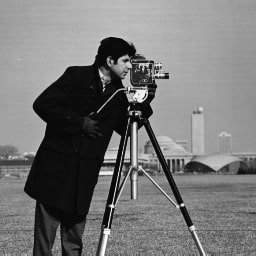

Supplement: Supplemental Information 2 — For Training purposes- Berkeley Segmentation dataset300 was used named soniya-mask. CBSD68, Set12, McMaster, and Kodak24 were used for testing purposes. [file peerj-cs-11-2449-s002.zip › overall database/Set12/01.jpg]

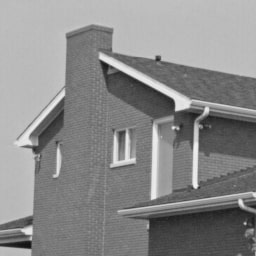

Supplement: Supplemental Information 2 — For Training purposes- Berkeley Segmentation dataset300 was used named soniya-mask. CBSD68, Set12, McMaster, and Kodak24 were used for testing purposes. [file peerj-cs-11-2449-s002.zip › overall database/Set12/02.jpg]

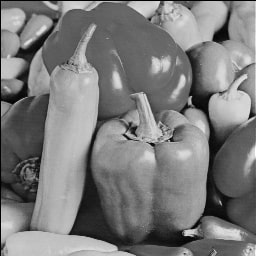

Supplement: Supplemental Information 2 — For Training purposes- Berkeley Segmentation dataset300 was used named soniya-mask. CBSD68, Set12, McMaster, and Kodak24 were used for testing purposes. [file peerj-cs-11-2449-s002.zip › overall database/Set12/03.jpg]

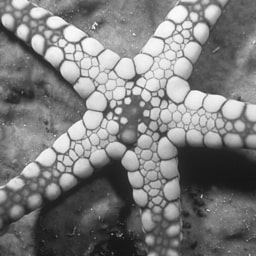

Supplement: Supplemental Information 2 — For Training purposes- Berkeley Segmentation dataset300 was used named soniya-mask. CBSD68, Set12, McMaster, and Kodak24 were used for testing purposes. [file peerj-cs-11-2449-s002.zip › overall database/Set12/04.jpg]

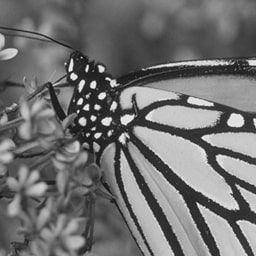

Supplement: Supplemental Information 2 — For Training purposes- Berkeley Segmentation dataset300 was used named soniya-mask. CBSD68, Set12, McMaster, and Kodak24 were used for testing purposes. [file peerj-cs-11-2449-s002.zip › overall database/Set12/05.jpg]

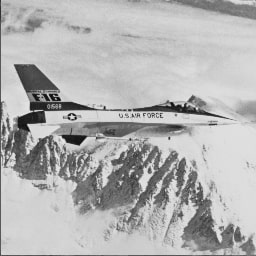

Supplement: Supplemental Information 2 — For Training purposes- Berkeley Segmentation dataset300 was used named soniya-mask. CBSD68, Set12, McMaster, and Kodak24 were used for testing purposes. [file peerj-cs-11-2449-s002.zip › overall database/Set12/06.jpg]

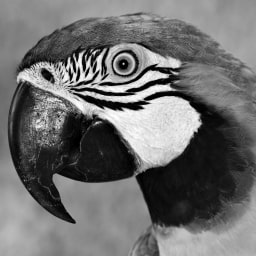

Supplement: Supplemental Information 2 — For Training purposes- Berkeley Segmentation dataset300 was used named soniya-mask. CBSD68, Set12, McMaster, and Kodak24 were used for testing purposes. [file peerj-cs-11-2449-s002.zip › overall database/Set12/07.jpg]

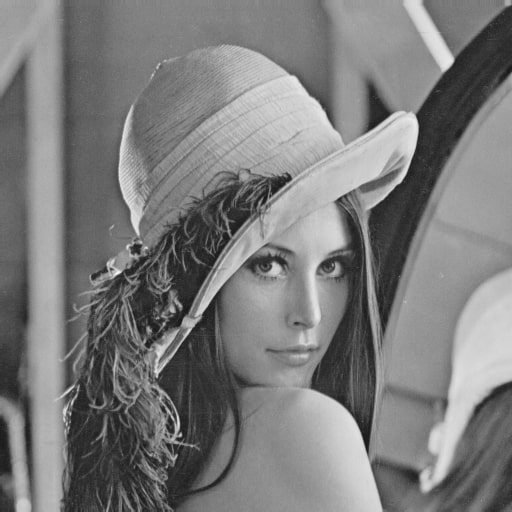

Supplement: Supplemental Information 2 — For Training purposes- Berkeley Segmentation dataset300 was used named soniya-mask. CBSD68, Set12, McMaster, and Kodak24 were used for testing purposes. [file peerj-cs-11-2449-s002.zip › overall database/Set12/08.jpg]

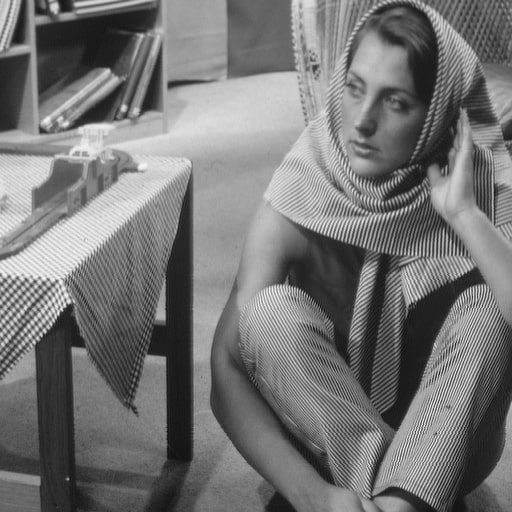

Supplement: Supplemental Information 2 — For Training purposes- Berkeley Segmentation dataset300 was used named soniya-mask. CBSD68, Set12, McMaster, and Kodak24 were used for testing purposes. [file peerj-cs-11-2449-s002.zip › overall database/Set12/09.jpg]

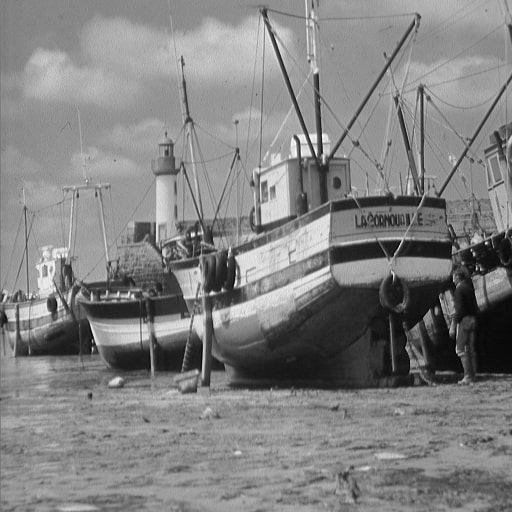

Supplement: Supplemental Information 2 — For Training purposes- Berkeley Segmentation dataset300 was used named soniya-mask. CBSD68, Set12, McMaster, and Kodak24 were used for testing purposes. [file peerj-cs-11-2449-s002.zip › overall database/Set12/10.jpg]

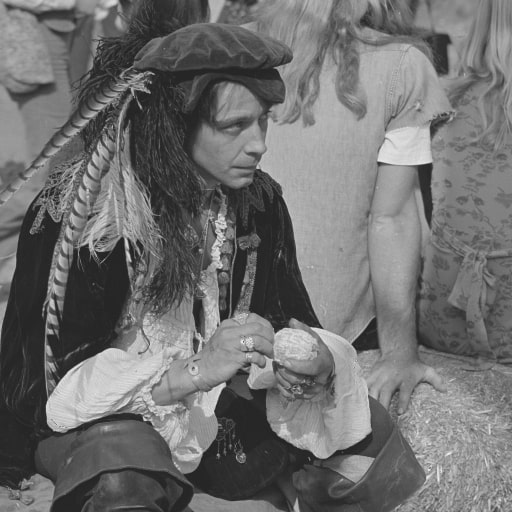

Supplement: Supplemental Information 2 — For Training purposes- Berkeley Segmentation dataset300 was used named soniya-mask. CBSD68, Set12, McMaster, and Kodak24 were used for testing purposes. [file peerj-cs-11-2449-s002.zip › overall database/Set12/11.jpg]

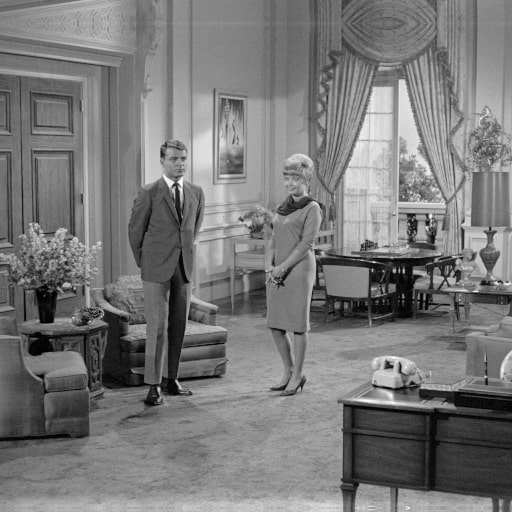

Supplement: Supplemental Information 2 — For Training purposes- Berkeley Segmentation dataset300 was used named soniya-mask. CBSD68, Set12, McMaster, and Kodak24 were used for testing purposes. [file peerj-cs-11-2449-s002.zip › overall database/Set12/12.jpg]

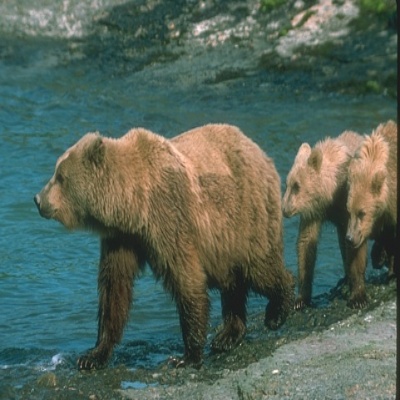

Supplement: Supplemental Information 2 — For Training purposes- Berkeley Segmentation dataset300 was used named soniya-mask. CBSD68, Set12, McMaster, and Kodak24 were used for testing purposes. [file peerj-cs-11-2449-s002.zip › overall database/soniya_mask/100075.jpg]

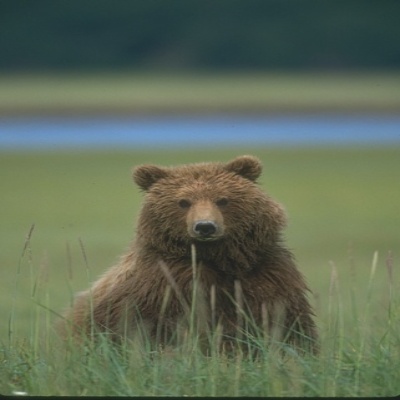

Supplement: Supplemental Information 2 — For Training purposes- Berkeley Segmentation dataset300 was used named soniya-mask. CBSD68, Set12, McMaster, and Kodak24 were used for testing purposes. [file peerj-cs-11-2449-s002.zip › overall database/soniya_mask/100080.jpg]

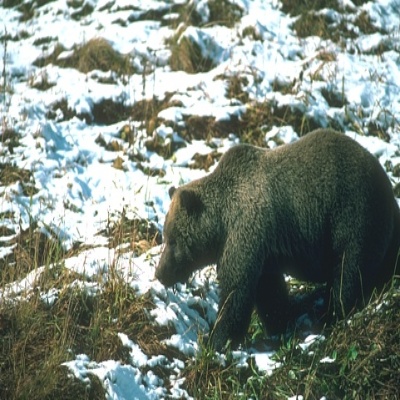

Supplement: Supplemental Information 2 — For Training purposes- Berkeley Segmentation dataset300 was used named soniya-mask. CBSD68, Set12, McMaster, and Kodak24 were used for testing purposes. [file peerj-cs-11-2449-s002.zip › overall database/soniya_mask/100098.jpg]

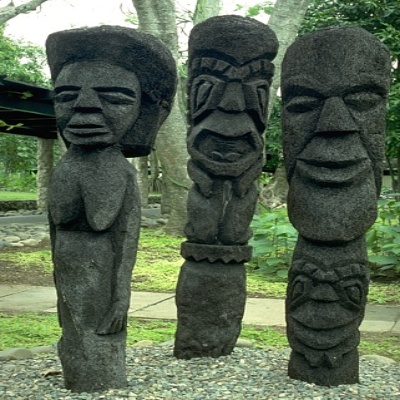

Supplement: Supplemental Information 2 — For Training purposes- Berkeley Segmentation dataset300 was used named soniya-mask. CBSD68, Set12, McMaster, and Kodak24 were used for testing purposes. [file peerj-cs-11-2449-s002.zip › overall database/soniya_mask/101085.jpg]

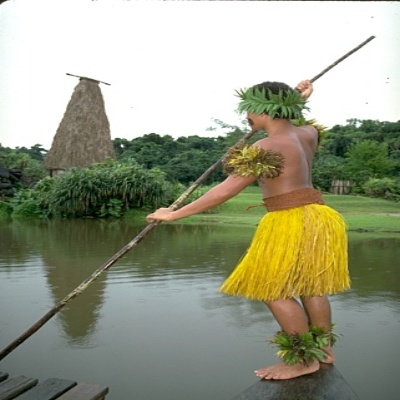

Supplement: Supplemental Information 2 — For Training purposes- Berkeley Segmentation dataset300 was used named soniya-mask. CBSD68, Set12, McMaster, and Kodak24 were used for testing purposes. [file peerj-cs-11-2449-s002.zip › overall database/soniya_mask/101087.jpg]

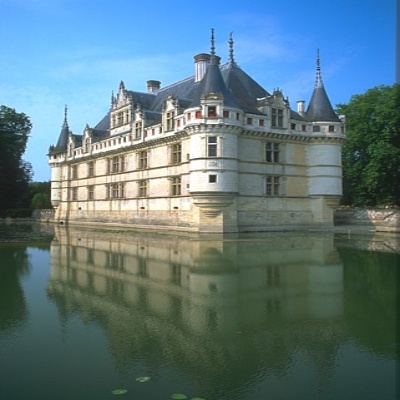

Supplement: Supplemental Information 2 — For Training purposes- Berkeley Segmentation dataset300 was used named soniya-mask. CBSD68, Set12, McMaster, and Kodak24 were used for testing purposes. [file peerj-cs-11-2449-s002.zip › overall database/soniya_mask/102061.jpg]

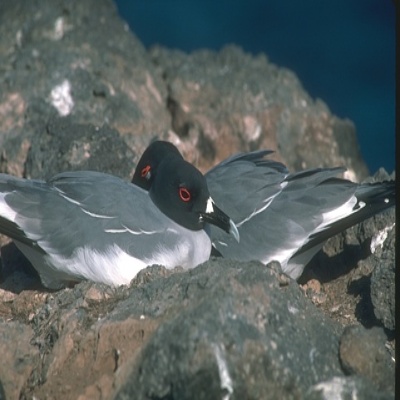

Supplement: Supplemental Information 2 — For Training purposes- Berkeley Segmentation dataset300 was used named soniya-mask. CBSD68, Set12, McMaster, and Kodak24 were used for testing purposes. [file peerj-cs-11-2449-s002.zip › overall database/soniya_mask/103041.jpg]

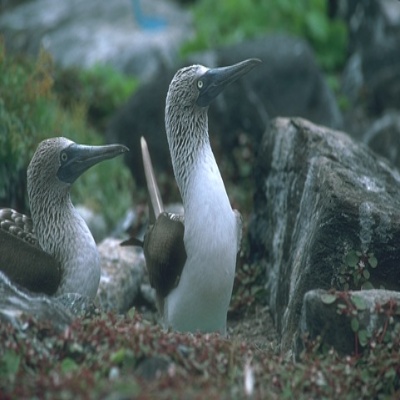

Supplement: Supplemental Information 2 — For Training purposes- Berkeley Segmentation dataset300 was used named soniya-mask. CBSD68, Set12, McMaster, and Kodak24 were used for testing purposes. [file peerj-cs-11-2449-s002.zip › overall database/soniya_mask/103070.jpg]

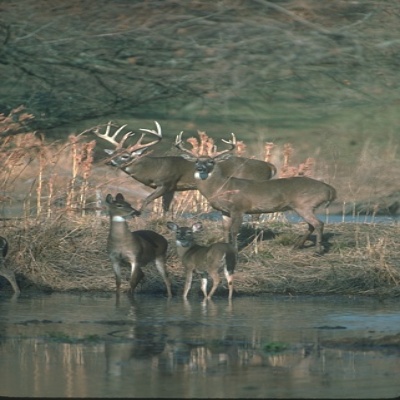

Supplement: Supplemental Information 2 — For Training purposes- Berkeley Segmentation dataset300 was used named soniya-mask. CBSD68, Set12, McMaster, and Kodak24 were used for testing purposes. [file peerj-cs-11-2449-s002.zip › overall database/soniya_mask/104022.jpg]

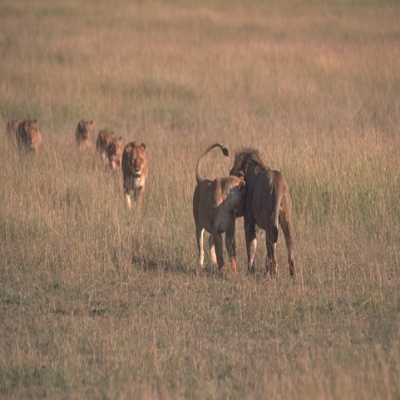

Supplement: Supplemental Information 2 — For Training purposes- Berkeley Segmentation dataset300 was used named soniya-mask. CBSD68, Set12, McMaster, and Kodak24 were used for testing purposes. [file peerj-cs-11-2449-s002.zip › overall database/soniya_mask/105019.jpg]

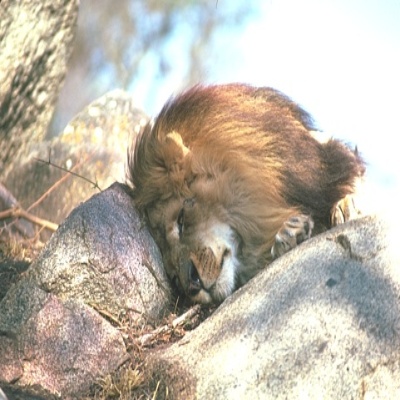

Supplement: Supplemental Information 2 — For Training purposes- Berkeley Segmentation dataset300 was used named soniya-mask. CBSD68, Set12, McMaster, and Kodak24 were used for testing purposes. [file peerj-cs-11-2449-s002.zip › overall database/soniya_mask/105025.jpg]

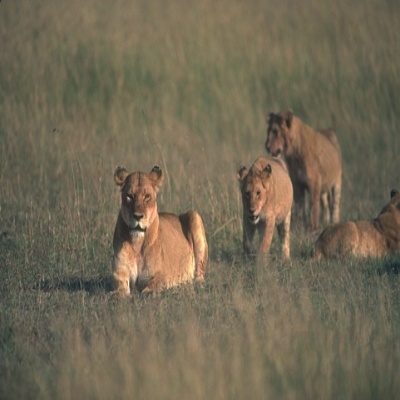

Supplement: Supplemental Information 2 — For Training purposes- Berkeley Segmentation dataset300 was used named soniya-mask. CBSD68, Set12, McMaster, and Kodak24 were used for testing purposes. [file peerj-cs-11-2449-s002.zip › overall database/soniya_mask/105053.jpg]

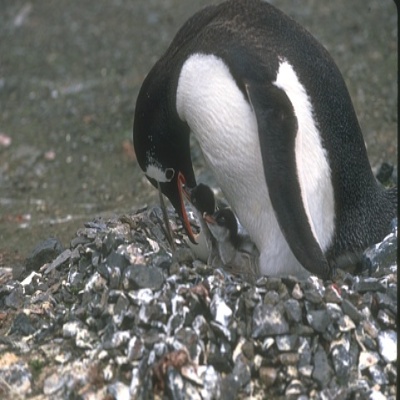

Supplement: Supplemental Information 2 — For Training purposes- Berkeley Segmentation dataset300 was used named soniya-mask. CBSD68, Set12, McMaster, and Kodak24 were used for testing purposes. [file peerj-cs-11-2449-s002.zip › overall database/soniya_mask/106020.jpg]

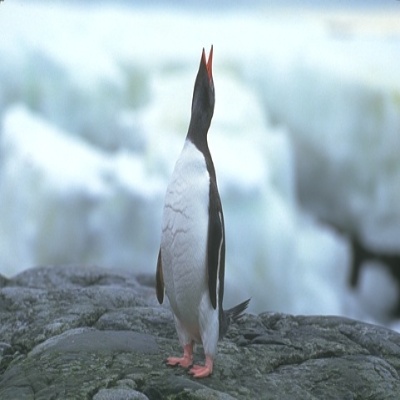

Supplement: Supplemental Information 2 — For Training purposes- Berkeley Segmentation dataset300 was used named soniya-mask. CBSD68, Set12, McMaster, and Kodak24 were used for testing purposes. [file peerj-cs-11-2449-s002.zip › overall database/soniya_mask/106024.jpg]

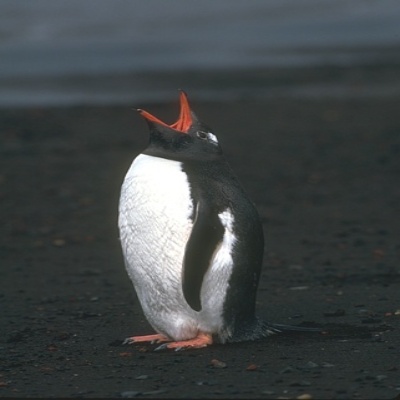

Supplement: Supplemental Information 2 — For Training purposes- Berkeley Segmentation dataset300 was used named soniya-mask. CBSD68, Set12, McMaster, and Kodak24 were used for testing purposes. [file peerj-cs-11-2449-s002.zip › overall database/soniya_mask/106025.jpg]

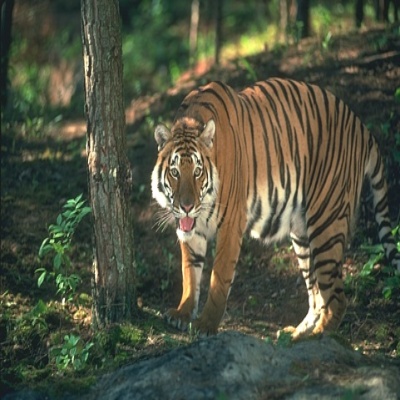

Supplement: Supplemental Information 2 — For Training purposes- Berkeley Segmentation dataset300 was used named soniya-mask. CBSD68, Set12, McMaster, and Kodak24 were used for testing purposes. [file peerj-cs-11-2449-s002.zip › overall database/soniya_mask/108005.jpg]

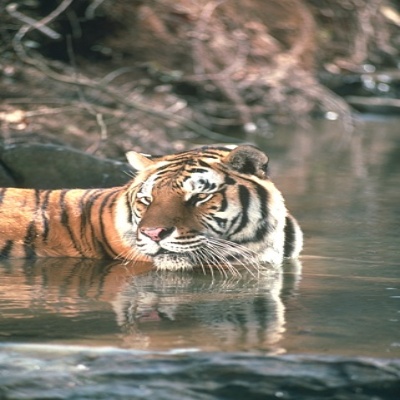

Supplement: Supplemental Information 2 — For Training purposes- Berkeley Segmentation dataset300 was used named soniya-mask. CBSD68, Set12, McMaster, and Kodak24 were used for testing purposes. [file peerj-cs-11-2449-s002.zip › overall database/soniya_mask/108041.jpg]

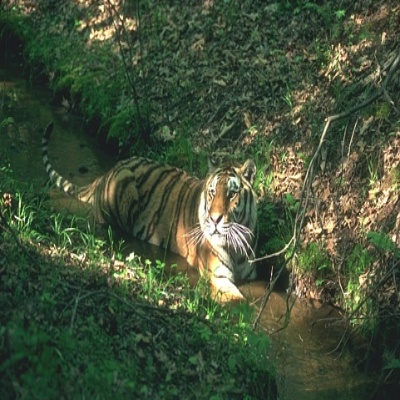

Supplement: Supplemental Information 2 — For Training purposes- Berkeley Segmentation dataset300 was used named soniya-mask. CBSD68, Set12, McMaster, and Kodak24 were used for testing purposes. [file peerj-cs-11-2449-s002.zip › overall database/soniya_mask/108070.jpg]

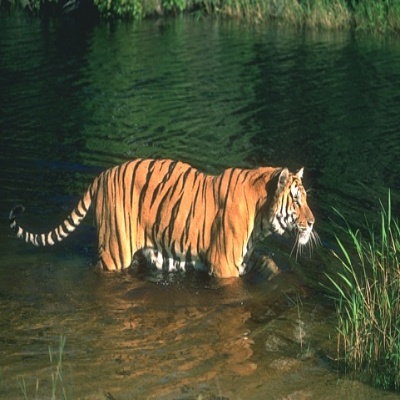

Supplement: Supplemental Information 2 — For Training purposes- Berkeley Segmentation dataset300 was used named soniya-mask. CBSD68, Set12, McMaster, and Kodak24 were used for testing purposes. [file peerj-cs-11-2449-s002.zip › overall database/soniya_mask/108073.jpg]

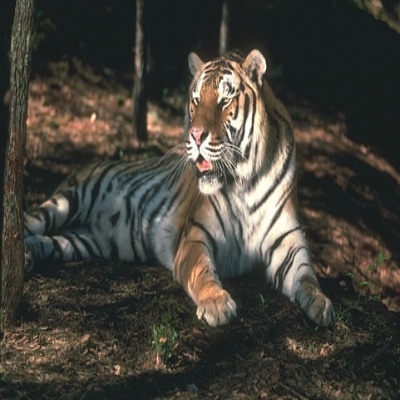

Supplement: Supplemental Information 2 — For Training purposes- Berkeley Segmentation dataset300 was used named soniya-mask. CBSD68, Set12, McMaster, and Kodak24 were used for testing purposes. [file peerj-cs-11-2449-s002.zip › overall database/soniya_mask/108082.jpg]

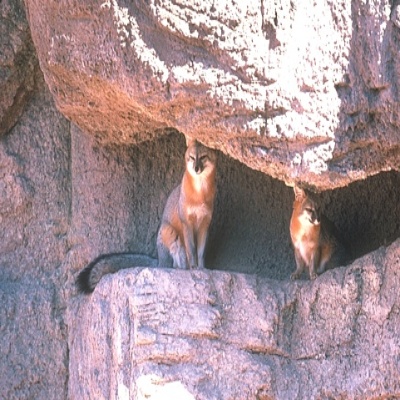

Supplement: Supplemental Information 2 — For Training purposes- Berkeley Segmentation dataset300 was used named soniya-mask. CBSD68, Set12, McMaster, and Kodak24 were used for testing purposes. [file peerj-cs-11-2449-s002.zip › overall database/soniya_mask/109034.jpg]

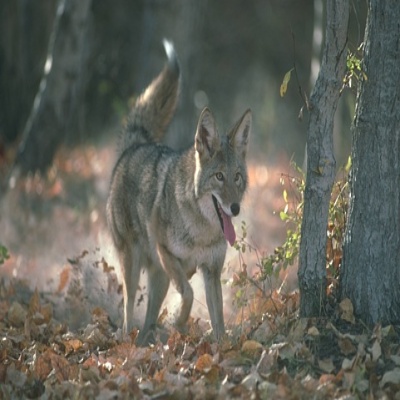

Supplement: Supplemental Information 2 — For Training purposes- Berkeley Segmentation dataset300 was used named soniya-mask. CBSD68, Set12, McMaster, and Kodak24 were used for testing purposes. [file peerj-cs-11-2449-s002.zip › overall database/soniya_mask/109053.jpg]

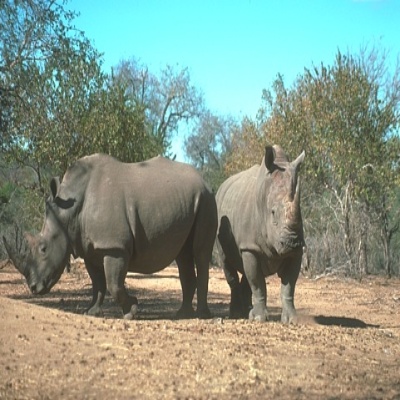

Supplement: Supplemental Information 2 — For Training purposes- Berkeley Segmentation dataset300 was used named soniya-mask. CBSD68, Set12, McMaster, and Kodak24 were used for testing purposes. [file peerj-cs-11-2449-s002.zip › overall database/soniya_mask/112082.jpg]

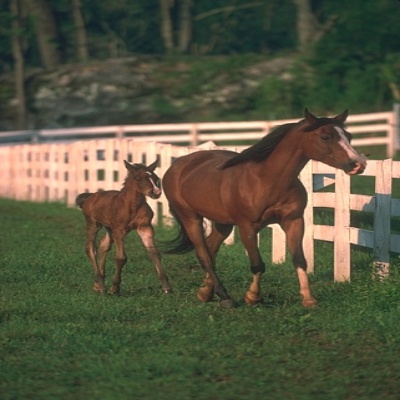

Supplement: Supplemental Information 2 — For Training purposes- Berkeley Segmentation dataset300 was used named soniya-mask. CBSD68, Set12, McMaster, and Kodak24 were used for testing purposes. [file peerj-cs-11-2449-s002.zip › overall database/soniya_mask/113009.jpg]

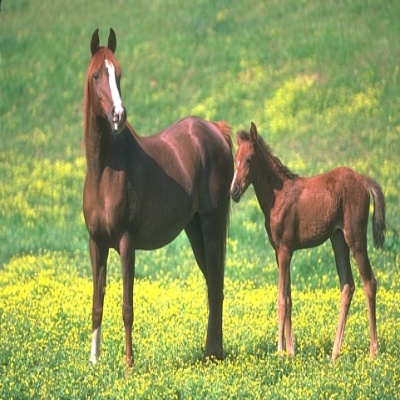

Supplement: Supplemental Information 2 — For Training purposes- Berkeley Segmentation dataset300 was used named soniya-mask. CBSD68, Set12, McMaster, and Kodak24 were used for testing purposes. [file peerj-cs-11-2449-s002.zip › overall database/soniya_mask/113016.jpg]

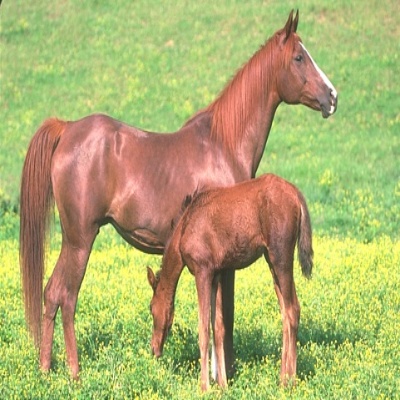

Supplement: Supplemental Information 2 — For Training purposes- Berkeley Segmentation dataset300 was used named soniya-mask. CBSD68, Set12, McMaster, and Kodak24 were used for testing purposes. [file peerj-cs-11-2449-s002.zip › overall database/soniya_mask/113044.jpg]

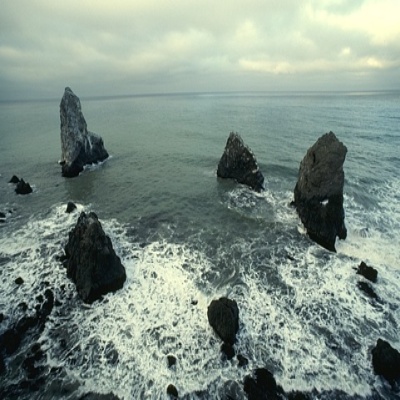

Supplement: Supplemental Information 2 — For Training purposes- Berkeley Segmentation dataset300 was used named soniya-mask. CBSD68, Set12, McMaster, and Kodak24 were used for testing purposes. [file peerj-cs-11-2449-s002.zip › overall database/soniya_mask/117054.jpg]

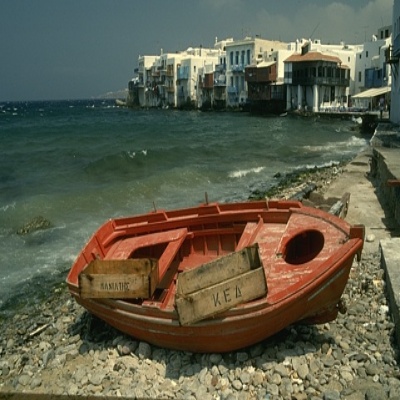

Supplement: Supplemental Information 2 — For Training purposes- Berkeley Segmentation dataset300 was used named soniya-mask. CBSD68, Set12, McMaster, and Kodak24 were used for testing purposes. [file peerj-cs-11-2449-s002.zip › overall database/soniya_mask/118020.jpg]

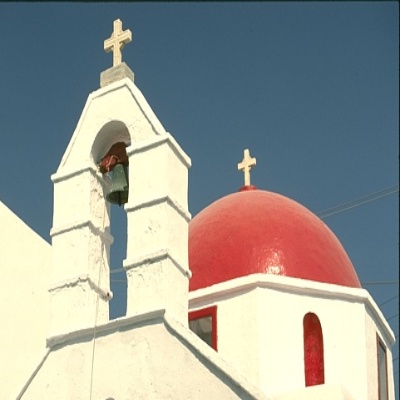

Supplement: Supplemental Information 2 — For Training purposes- Berkeley Segmentation dataset300 was used named soniya-mask. CBSD68, Set12, McMaster, and Kodak24 were used for testing purposes. [file peerj-cs-11-2449-s002.zip › overall database/soniya_mask/118035.jpg]

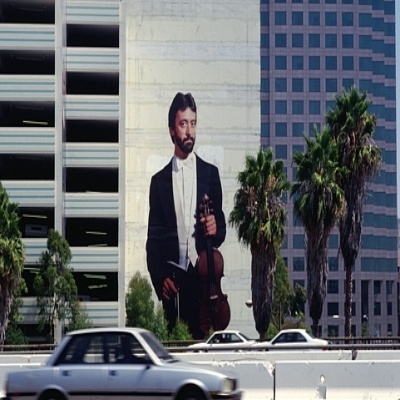

Supplement: Supplemental Information 2 — For Training purposes- Berkeley Segmentation dataset300 was used named soniya-mask. CBSD68, Set12, McMaster, and Kodak24 were used for testing purposes. [file peerj-cs-11-2449-s002.zip › overall database/soniya_mask/119082.jpg]

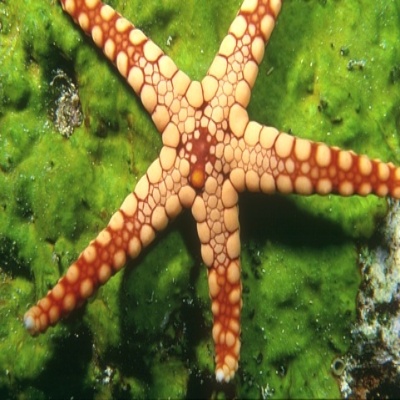

Supplement: Supplemental Information 2 — For Training purposes- Berkeley Segmentation dataset300 was used named soniya-mask. CBSD68, Set12, McMaster, and Kodak24 were used for testing purposes. [file peerj-cs-11-2449-s002.zip › overall database/soniya_mask/12003.jpg]

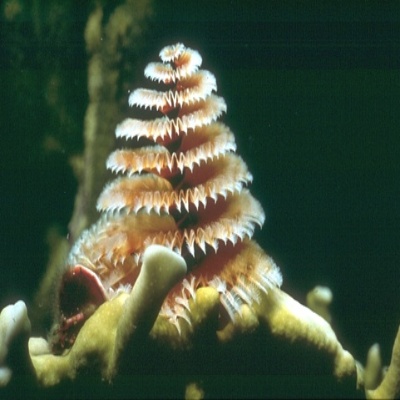

Supplement: Supplemental Information 2 — For Training purposes- Berkeley Segmentation dataset300 was used named soniya-mask. CBSD68, Set12, McMaster, and Kodak24 were used for testing purposes. [file peerj-cs-11-2449-s002.zip › overall database/soniya_mask/12074.jpg]

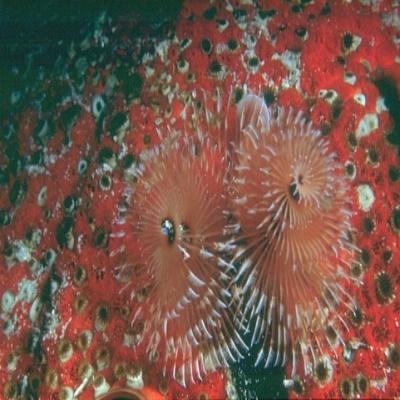

Supplement: Supplemental Information 2 — For Training purposes- Berkeley Segmentation dataset300 was used named soniya-mask. CBSD68, Set12, McMaster, and Kodak24 were used for testing purposes. [file peerj-cs-11-2449-s002.zip › overall database/soniya_mask/12084.jpg]

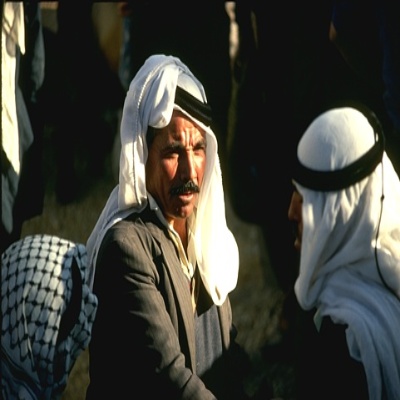

Supplement: Supplemental Information 2 — For Training purposes- Berkeley Segmentation dataset300 was used named soniya-mask. CBSD68, Set12, McMaster, and Kodak24 were used for testing purposes. [file peerj-cs-11-2449-s002.zip › overall database/soniya_mask/122048.jpg]

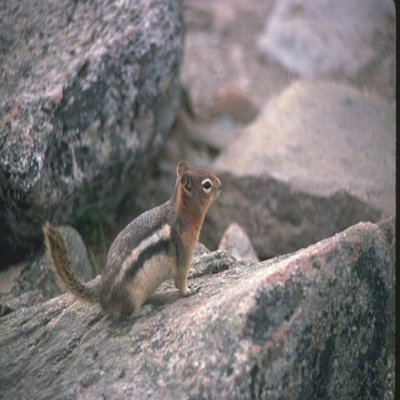

Supplement: Supplemental Information 2 — For Training purposes- Berkeley Segmentation dataset300 was used named soniya-mask. CBSD68, Set12, McMaster, and Kodak24 were used for testing purposes. [file peerj-cs-11-2449-s002.zip › overall database/soniya_mask/123074.jpg]

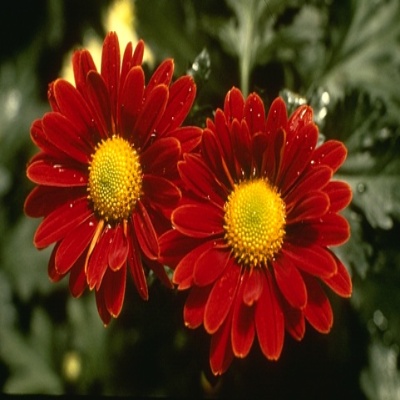

Supplement: Supplemental Information 2 — For Training purposes- Berkeley Segmentation dataset300 was used named soniya-mask. CBSD68, Set12, McMaster, and Kodak24 were used for testing purposes. [file peerj-cs-11-2449-s002.zip › overall database/soniya_mask/124084.jpg]

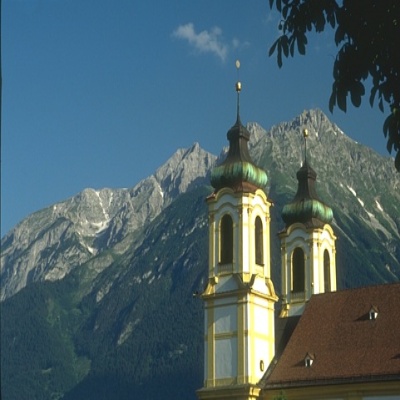

Supplement: Supplemental Information 2 — For Training purposes- Berkeley Segmentation dataset300 was used named soniya-mask. CBSD68, Set12, McMaster, and Kodak24 were used for testing purposes. [file peerj-cs-11-2449-s002.zip › overall database/soniya_mask/126007.jpg]

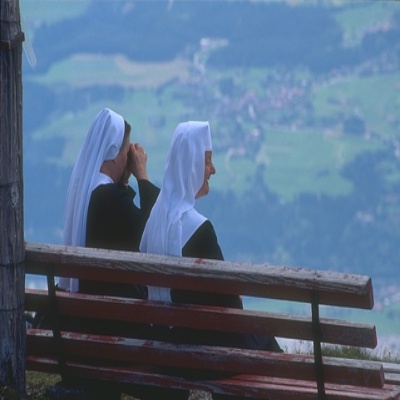

Supplement: Supplemental Information 2 — For Training purposes- Berkeley Segmentation dataset300 was used named soniya-mask. CBSD68, Set12, McMaster, and Kodak24 were used for testing purposes. [file peerj-cs-11-2449-s002.zip › overall database/soniya_mask/126039.jpg]

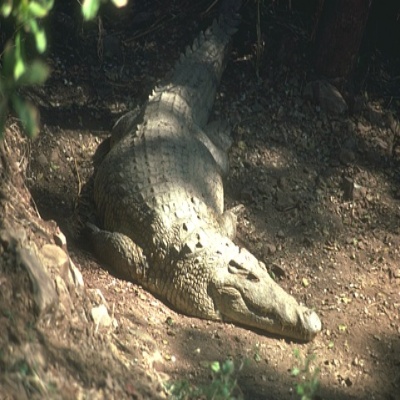

Supplement: Supplemental Information 2 — For Training purposes- Berkeley Segmentation dataset300 was used named soniya-mask. CBSD68, Set12, McMaster, and Kodak24 were used for testing purposes. [file peerj-cs-11-2449-s002.zip › overall database/soniya_mask/130026.jpg]

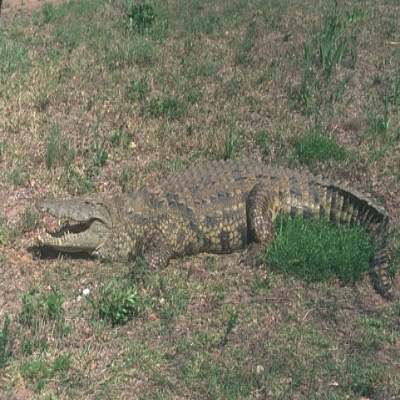

Supplement: Supplemental Information 2 — For Training purposes- Berkeley Segmentation dataset300 was used named soniya-mask. CBSD68, Set12, McMaster, and Kodak24 were used for testing purposes. [file peerj-cs-11-2449-s002.zip › overall database/soniya_mask/130034.jpg]

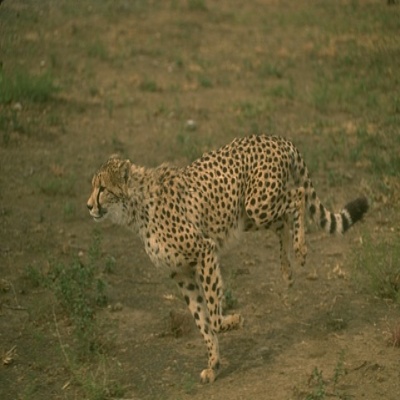

Supplement: Supplemental Information 2 — For Training purposes- Berkeley Segmentation dataset300 was used named soniya-mask. CBSD68, Set12, McMaster, and Kodak24 were used for testing purposes. [file peerj-cs-11-2449-s002.zip › overall database/soniya_mask/134008.jpg]

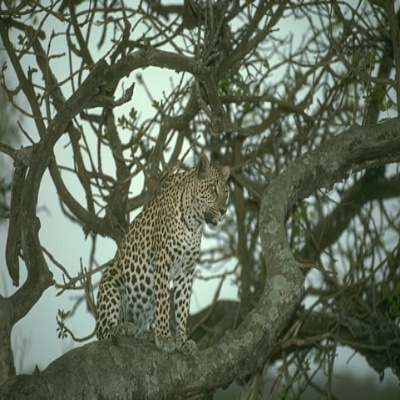

Supplement: Supplemental Information 2 — For Training purposes- Berkeley Segmentation dataset300 was used named soniya-mask. CBSD68, Set12, McMaster, and Kodak24 were used for testing purposes. [file peerj-cs-11-2449-s002.zip › overall database/soniya_mask/134035.jpg]

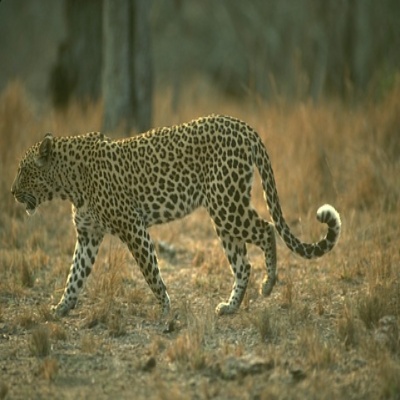

Supplement: Supplemental Information 2 — For Training purposes- Berkeley Segmentation dataset300 was used named soniya-mask. CBSD68, Set12, McMaster, and Kodak24 were used for testing purposes. [file peerj-cs-11-2449-s002.zip › overall database/soniya_mask/134052.jpg]

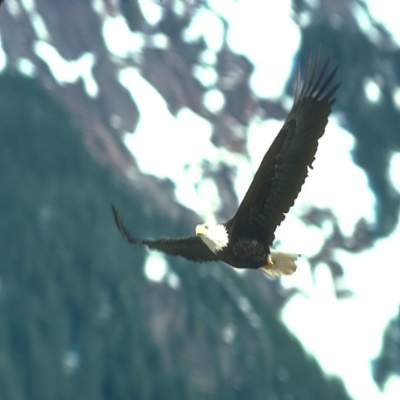

Supplement: Supplemental Information 2 — For Training purposes- Berkeley Segmentation dataset300 was used named soniya-mask. CBSD68, Set12, McMaster, and Kodak24 were used for testing purposes. [file peerj-cs-11-2449-s002.zip › overall database/soniya_mask/135037.jpg]

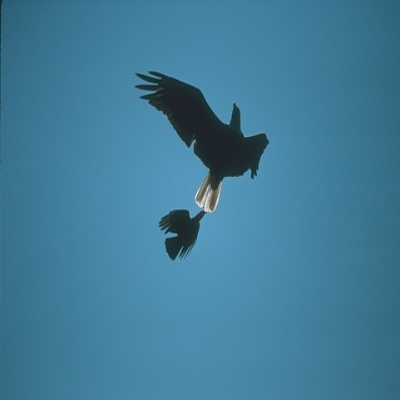

Supplement: Supplemental Information 2 — For Training purposes- Berkeley Segmentation dataset300 was used named soniya-mask. CBSD68, Set12, McMaster, and Kodak24 were used for testing purposes. [file peerj-cs-11-2449-s002.zip › overall database/soniya_mask/135069.jpg]

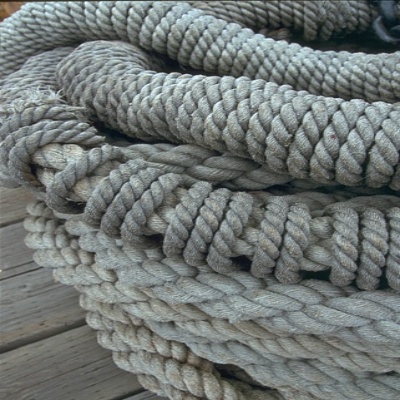

Supplement: Supplemental Information 2 — For Training purposes- Berkeley Segmentation dataset300 was used named soniya-mask. CBSD68, Set12, McMaster, and Kodak24 were used for testing purposes. [file peerj-cs-11-2449-s002.zip › overall database/soniya_mask/138032.jpg]

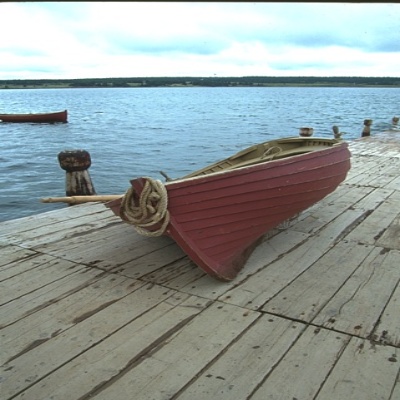

Supplement: Supplemental Information 2 — For Training purposes- Berkeley Segmentation dataset300 was used named soniya-mask. CBSD68, Set12, McMaster, and Kodak24 were used for testing purposes. [file peerj-cs-11-2449-s002.zip › overall database/soniya_mask/138078.jpg]

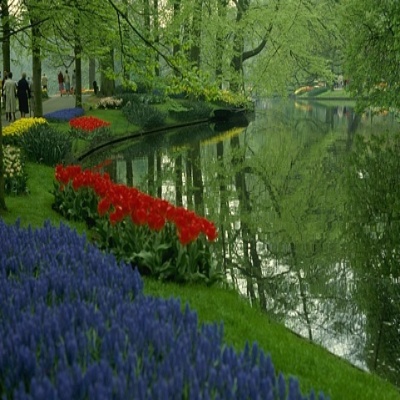

Supplement: Supplemental Information 2 — For Training purposes- Berkeley Segmentation dataset300 was used named soniya-mask. CBSD68, Set12, McMaster, and Kodak24 were used for testing purposes. [file peerj-cs-11-2449-s002.zip › overall database/soniya_mask/140055.jpg]

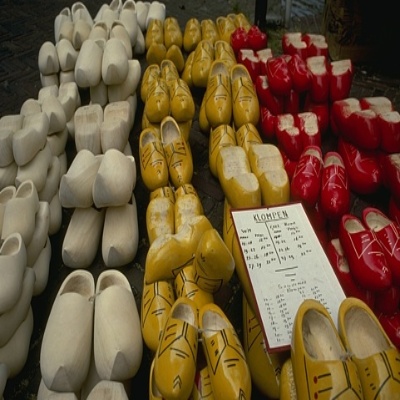

Supplement: Supplemental Information 2 — For Training purposes- Berkeley Segmentation dataset300 was used named soniya-mask. CBSD68, Set12, McMaster, and Kodak24 were used for testing purposes. [file peerj-cs-11-2449-s002.zip › overall database/soniya_mask/140075.jpg]

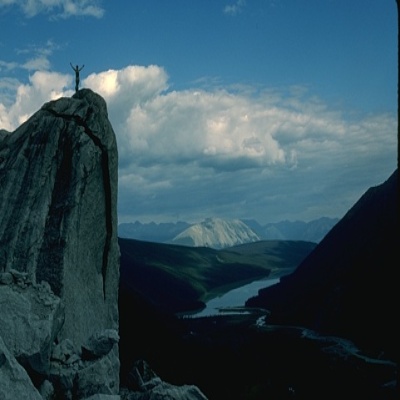

Supplement: Supplemental Information 2 — For Training purposes- Berkeley Segmentation dataset300 was used named soniya-mask. CBSD68, Set12, McMaster, and Kodak24 were used for testing purposes. [file peerj-cs-11-2449-s002.zip › overall database/soniya_mask/14037.jpg]

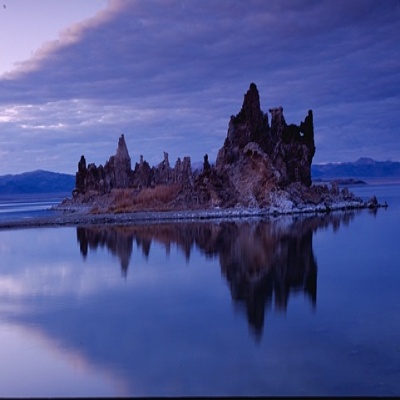

Supplement: Supplemental Information 2 — For Training purposes- Berkeley Segmentation dataset300 was used named soniya-mask. CBSD68, Set12, McMaster, and Kodak24 were used for testing purposes. [file peerj-cs-11-2449-s002.zip › overall database/soniya_mask/143090.jpg]

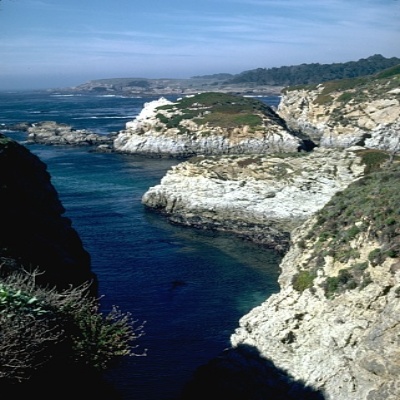

Supplement: Supplemental Information 2 — For Training purposes- Berkeley Segmentation dataset300 was used named soniya-mask. CBSD68, Set12, McMaster, and Kodak24 were used for testing purposes. [file peerj-cs-11-2449-s002.zip › overall database/soniya_mask/144067.jpg]

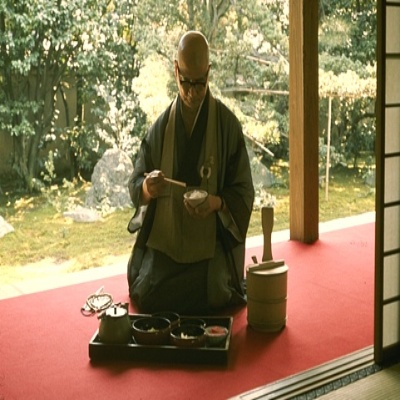

Supplement: Supplemental Information 2 — For Training purposes- Berkeley Segmentation dataset300 was used named soniya-mask. CBSD68, Set12, McMaster, and Kodak24 were used for testing purposes. [file peerj-cs-11-2449-s002.zip › overall database/soniya_mask/145014.jpg]

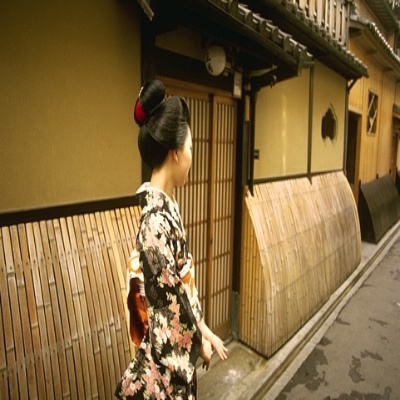

Supplement: Supplemental Information 2 — For Training purposes- Berkeley Segmentation dataset300 was used named soniya-mask. CBSD68, Set12, McMaster, and Kodak24 were used for testing purposes. [file peerj-cs-11-2449-s002.zip › overall database/soniya_mask/145053.jpg]

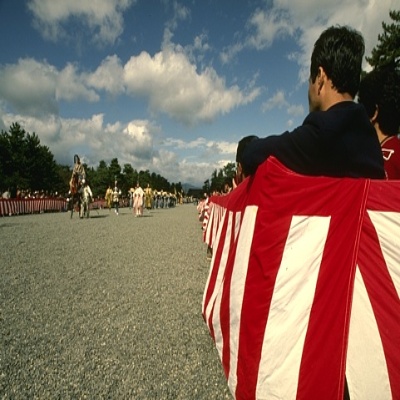

Supplement: Supplemental Information 2 — For Training purposes- Berkeley Segmentation dataset300 was used named soniya-mask. CBSD68, Set12, McMaster, and Kodak24 were used for testing purposes. [file peerj-cs-11-2449-s002.zip › overall database/soniya_mask/145086.jpg]

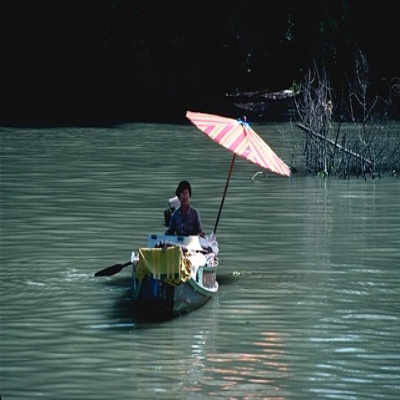

Supplement: Supplemental Information 2 — For Training purposes- Berkeley Segmentation dataset300 was used named soniya-mask. CBSD68, Set12, McMaster, and Kodak24 were used for testing purposes. [file peerj-cs-11-2449-s002.zip › overall database/soniya_mask/147021.jpg]

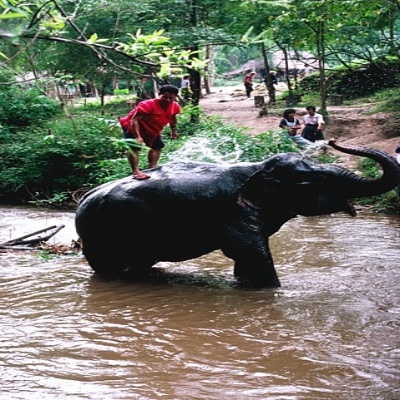

Supplement: Supplemental Information 2 — For Training purposes- Berkeley Segmentation dataset300 was used named soniya-mask. CBSD68, Set12, McMaster, and Kodak24 were used for testing purposes. [file peerj-cs-11-2449-s002.zip › overall database/soniya_mask/147062.jpg]

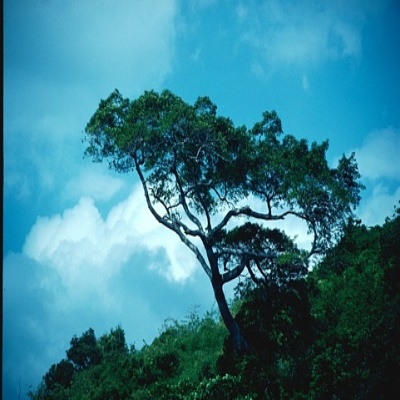

Supplement: Supplemental Information 2 — For Training purposes- Berkeley Segmentation dataset300 was used named soniya-mask. CBSD68, Set12, McMaster, and Kodak24 were used for testing purposes. [file peerj-cs-11-2449-s002.zip › overall database/soniya_mask/147091.jpg]

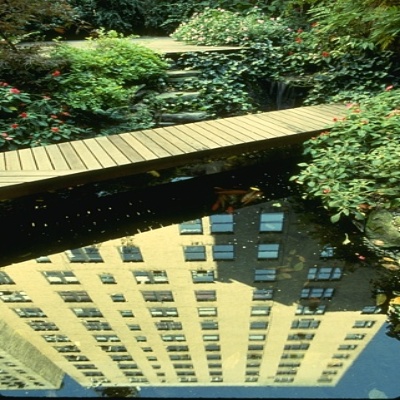

Supplement: Supplemental Information 2 — For Training purposes- Berkeley Segmentation dataset300 was used named soniya-mask. CBSD68, Set12, McMaster, and Kodak24 were used for testing purposes. [file peerj-cs-11-2449-s002.zip › overall database/soniya_mask/148026.jpg]

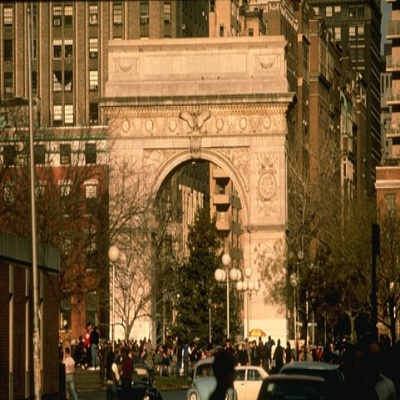

Supplement: Supplemental Information 2 — For Training purposes- Berkeley Segmentation dataset300 was used named soniya-mask. CBSD68, Set12, McMaster, and Kodak24 were used for testing purposes. [file peerj-cs-11-2449-s002.zip › overall database/soniya_mask/148089.jpg]

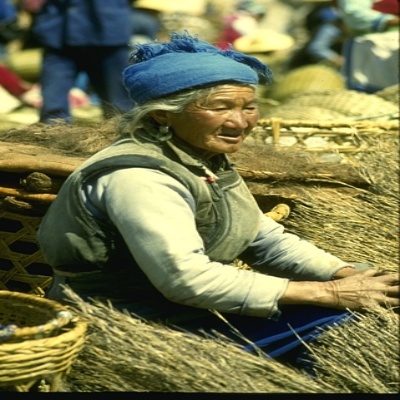

Supplement: Supplemental Information 2 — For Training purposes- Berkeley Segmentation dataset300 was used named soniya-mask. CBSD68, Set12, McMaster, and Kodak24 were used for testing purposes. [file peerj-cs-11-2449-s002.zip › overall database/soniya_mask/15004.jpg]

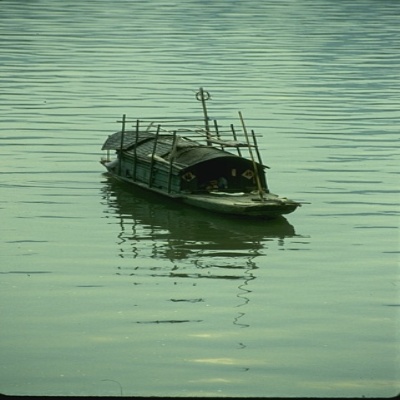

Supplement: Supplemental Information 2 — For Training purposes- Berkeley Segmentation dataset300 was used named soniya-mask. CBSD68, Set12, McMaster, and Kodak24 were used for testing purposes. [file peerj-cs-11-2449-s002.zip › overall database/soniya_mask/15088.jpg]

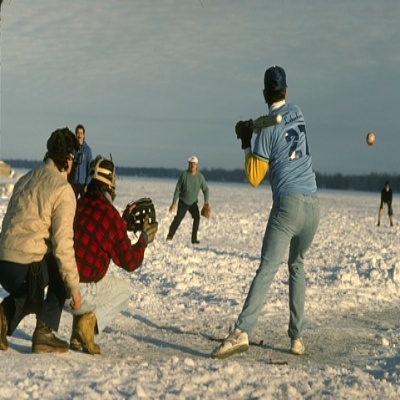

Supplement: Supplemental Information 2 — For Training purposes- Berkeley Segmentation dataset300 was used named soniya-mask. CBSD68, Set12, McMaster, and Kodak24 were used for testing purposes. [file peerj-cs-11-2449-s002.zip › overall database/soniya_mask/151087.jpg]

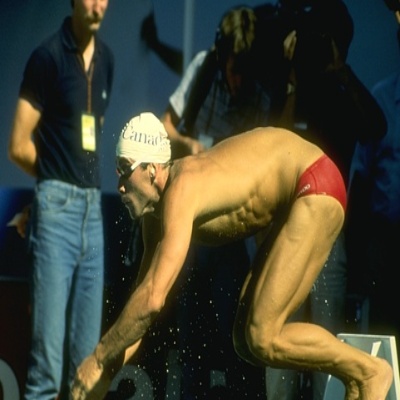

Supplement: Supplemental Information 2 — For Training purposes- Berkeley Segmentation dataset300 was used named soniya-mask. CBSD68, Set12, McMaster, and Kodak24 were used for testing purposes. [file peerj-cs-11-2449-s002.zip › overall database/soniya_mask/153077.jpg]

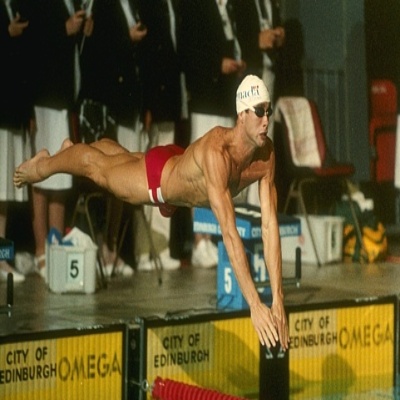

Supplement: Supplemental Information 2 — For Training purposes- Berkeley Segmentation dataset300 was used named soniya-mask. CBSD68, Set12, McMaster, and Kodak24 were used for testing purposes. [file peerj-cs-11-2449-s002.zip › overall database/soniya_mask/153093.jpg]

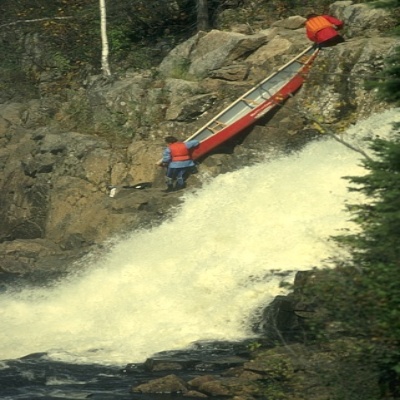

Supplement: Supplemental Information 2 — For Training purposes- Berkeley Segmentation dataset300 was used named soniya-mask. CBSD68, Set12, McMaster, and Kodak24 were used for testing purposes. [file peerj-cs-11-2449-s002.zip › overall database/soniya_mask/155060.jpg]

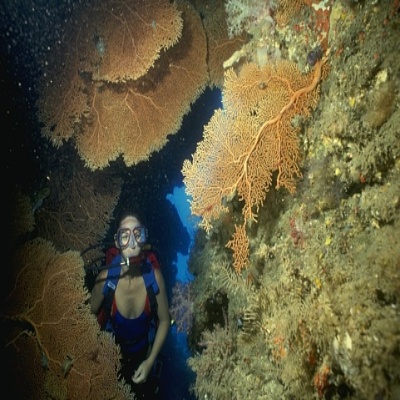

Supplement: Supplemental Information 2 — For Training purposes- Berkeley Segmentation dataset300 was used named soniya-mask. CBSD68, Set12, McMaster, and Kodak24 were used for testing purposes. [file peerj-cs-11-2449-s002.zip › overall database/soniya_mask/156065.jpg]

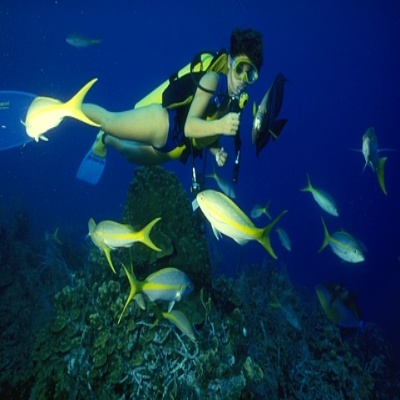

Supplement: Supplemental Information 2 — For Training purposes- Berkeley Segmentation dataset300 was used named soniya-mask. CBSD68, Set12, McMaster, and Kodak24 were used for testing purposes. [file peerj-cs-11-2449-s002.zip › overall database/soniya_mask/156079.jpg]

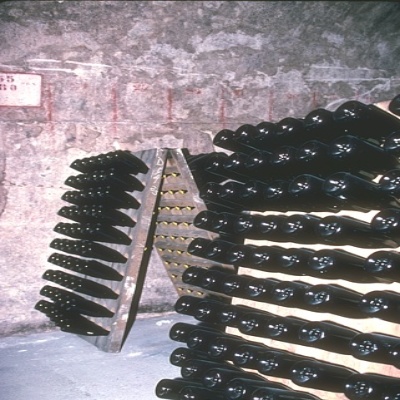

Supplement: Supplemental Information 2 — For Training purposes- Berkeley Segmentation dataset300 was used named soniya-mask. CBSD68, Set12, McMaster, and Kodak24 were used for testing purposes. [file peerj-cs-11-2449-s002.zip › overall database/soniya_mask/157036.jpg]

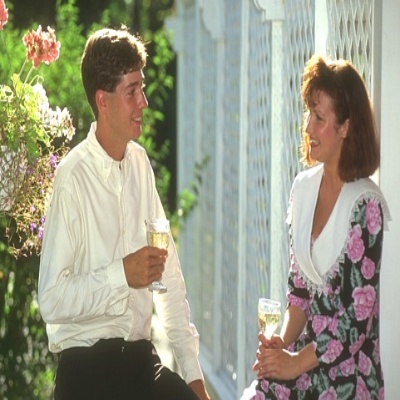

Supplement: Supplemental Information 2 — For Training purposes- Berkeley Segmentation dataset300 was used named soniya-mask. CBSD68, Set12, McMaster, and Kodak24 were used for testing purposes. [file peerj-cs-11-2449-s002.zip › overall database/soniya_mask/157055.jpg]

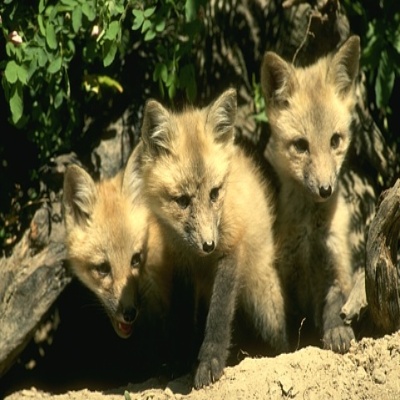

Supplement: Supplemental Information 2 — For Training purposes- Berkeley Segmentation dataset300 was used named soniya-mask. CBSD68, Set12, McMaster, and Kodak24 were used for testing purposes. [file peerj-cs-11-2449-s002.zip › overall database/soniya_mask/159008.jpg]

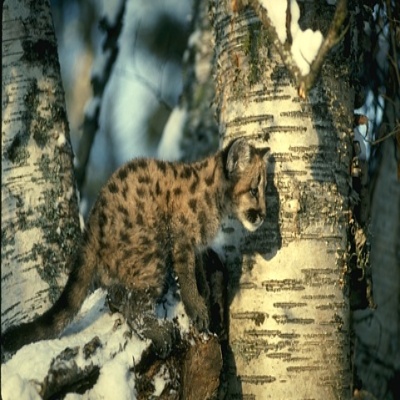

Supplement: Supplemental Information 2 — For Training purposes- Berkeley Segmentation dataset300 was used named soniya-mask. CBSD68, Set12, McMaster, and Kodak24 were used for testing purposes. [file peerj-cs-11-2449-s002.zip › overall database/soniya_mask/159029.jpg]

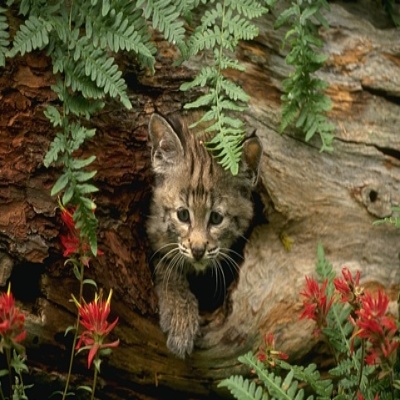

Supplement: Supplemental Information 2 — For Training purposes- Berkeley Segmentation dataset300 was used named soniya-mask. CBSD68, Set12, McMaster, and Kodak24 were used for testing purposes. [file peerj-cs-11-2449-s002.zip › overall database/soniya_mask/159045.jpg]

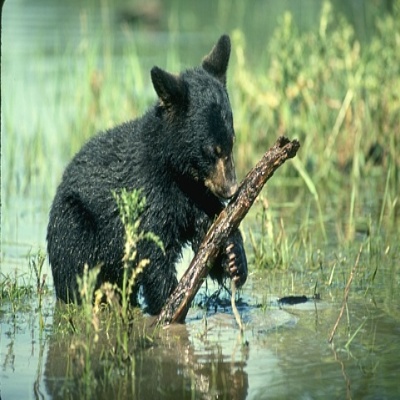

Supplement: Supplemental Information 2 — For Training purposes- Berkeley Segmentation dataset300 was used named soniya-mask. CBSD68, Set12, McMaster, and Kodak24 were used for testing purposes. [file peerj-cs-11-2449-s002.zip › overall database/soniya_mask/159091.jpg]

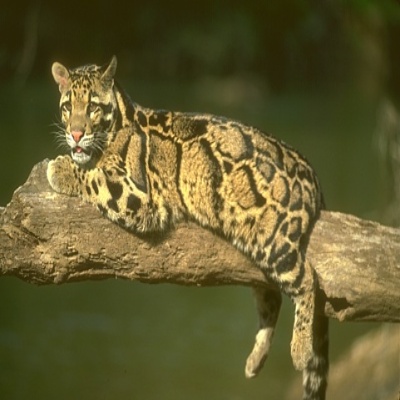

Supplement: Supplemental Information 2 — For Training purposes- Berkeley Segmentation dataset300 was used named soniya-mask. CBSD68, Set12, McMaster, and Kodak24 were used for testing purposes. [file peerj-cs-11-2449-s002.zip › overall database/soniya_mask/160068.jpg]

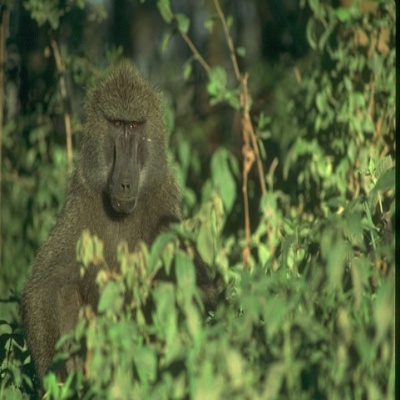

Supplement: Supplemental Information 2 — For Training purposes- Berkeley Segmentation dataset300 was used named soniya-mask. CBSD68, Set12, McMaster, and Kodak24 were used for testing purposes. [file peerj-cs-11-2449-s002.zip › overall database/soniya_mask/16052.jpg]

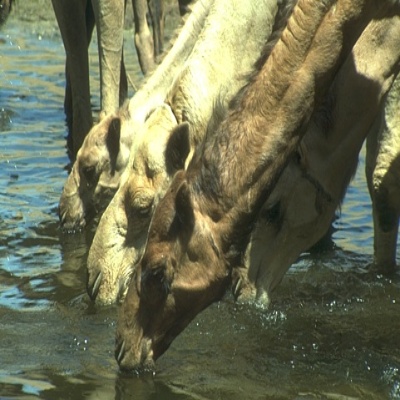

Supplement: Supplemental Information 2 — For Training purposes- Berkeley Segmentation dataset300 was used named soniya-mask. CBSD68, Set12, McMaster, and Kodak24 were used for testing purposes. [file peerj-cs-11-2449-s002.zip › overall database/soniya_mask/16077.jpg]

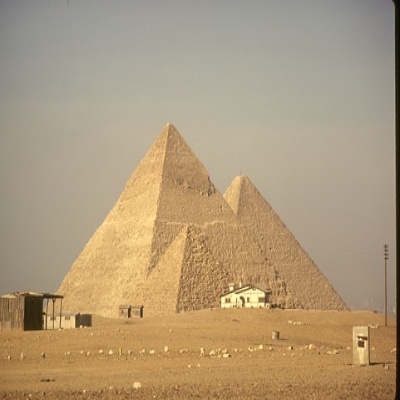

Supplement: Supplemental Information 2 — For Training purposes- Berkeley Segmentation dataset300 was used named soniya-mask. CBSD68, Set12, McMaster, and Kodak24 were used for testing purposes. [file peerj-cs-11-2449-s002.zip › overall database/soniya_mask/161062.jpg]
