# Supplementary figures and images for: Edge and texture aware image denoising using median noise residue U-net with hand-crafted features (part 1 of 2)
Source: PeerJ Comput Sci. 2025 Jan 16;11:e2449. doi: 10.7717/peerj-cs.2449 (PMC11784896; doi:10.7717/peerj-cs.2449)

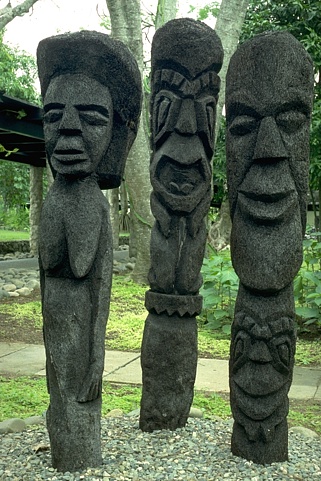

Supplement: Supplemental Information 2 — For Training purposes- Berkeley Segmentation dataset300 was used named soniya-mask. CBSD68, Set12, McMaster, and Kodak24 were used for testing purposes. [file peerj-cs-11-2449-s002.zip › overall database/CBSD68/101085.jpg]

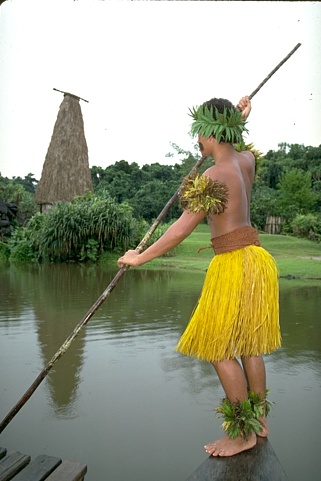

Supplement: Supplemental Information 2 — For Training purposes- Berkeley Segmentation dataset300 was used named soniya-mask. CBSD68, Set12, McMaster, and Kodak24 were used for testing purposes. [file peerj-cs-11-2449-s002.zip › overall database/CBSD68/101087.jpg]

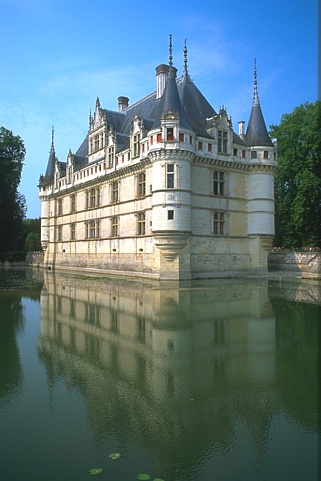

Supplement: Supplemental Information 2 — For Training purposes- Berkeley Segmentation dataset300 was used named soniya-mask. CBSD68, Set12, McMaster, and Kodak24 were used for testing purposes. [file peerj-cs-11-2449-s002.zip › overall database/CBSD68/102061.jpg]

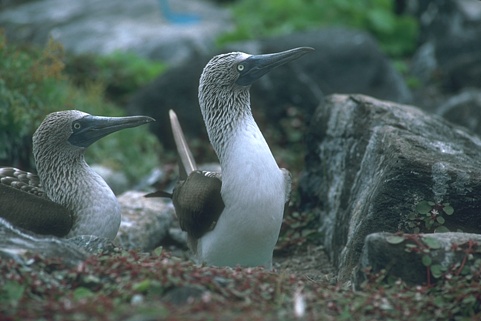

Supplement: Supplemental Information 2 — For Training purposes- Berkeley Segmentation dataset300 was used named soniya-mask. CBSD68, Set12, McMaster, and Kodak24 were used for testing purposes. [file peerj-cs-11-2449-s002.zip › overall database/CBSD68/103070.jpg]

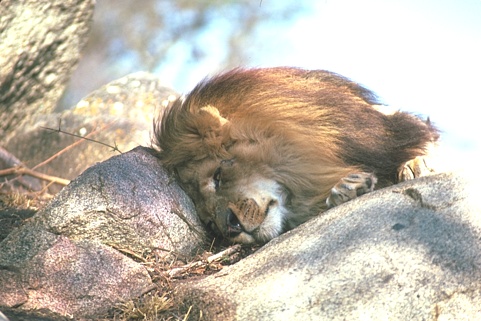

Supplement: Supplemental Information 2 — For Training purposes- Berkeley Segmentation dataset300 was used named soniya-mask. CBSD68, Set12, McMaster, and Kodak24 were used for testing purposes. [file peerj-cs-11-2449-s002.zip › overall database/CBSD68/105025.jpg]

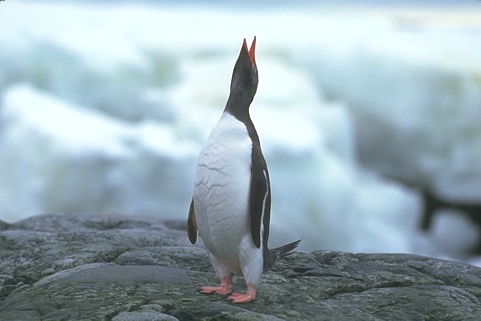

Supplement: Supplemental Information 2 — For Training purposes- Berkeley Segmentation dataset300 was used named soniya-mask. CBSD68, Set12, McMaster, and Kodak24 were used for testing purposes. [file peerj-cs-11-2449-s002.zip › overall database/CBSD68/106024.jpg]

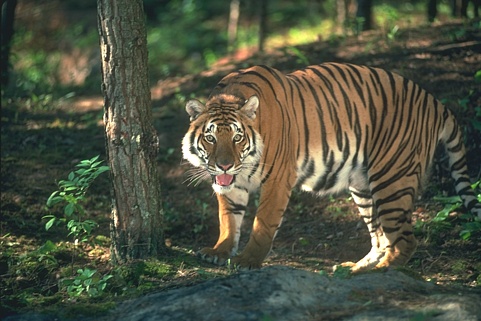

Supplement: Supplemental Information 2 — For Training purposes- Berkeley Segmentation dataset300 was used named soniya-mask. CBSD68, Set12, McMaster, and Kodak24 were used for testing purposes. [file peerj-cs-11-2449-s002.zip › overall database/CBSD68/108005.jpg]

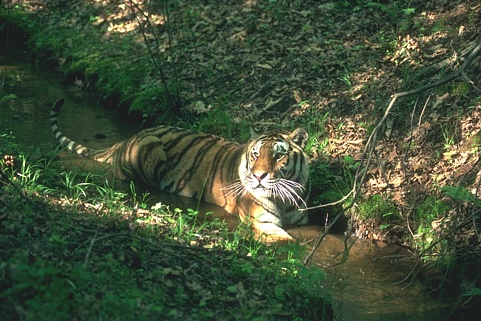

Supplement: Supplemental Information 2 — For Training purposes- Berkeley Segmentation dataset300 was used named soniya-mask. CBSD68, Set12, McMaster, and Kodak24 were used for testing purposes. [file peerj-cs-11-2449-s002.zip › overall database/CBSD68/108070.jpg]

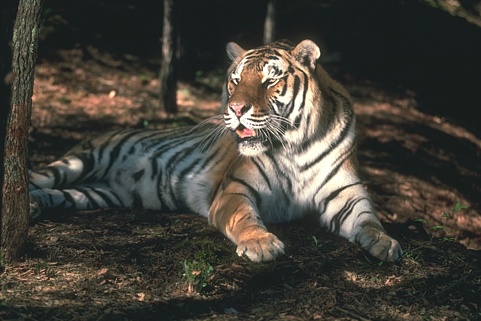

Supplement: Supplemental Information 2 — For Training purposes- Berkeley Segmentation dataset300 was used named soniya-mask. CBSD68, Set12, McMaster, and Kodak24 were used for testing purposes. [file peerj-cs-11-2449-s002.zip › overall database/CBSD68/108082.jpg]

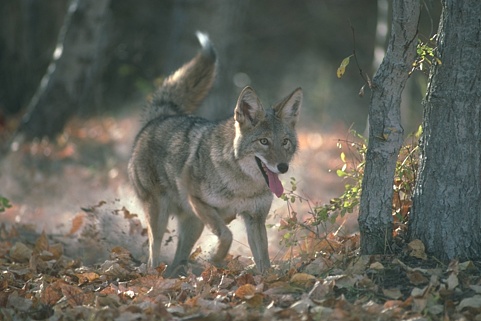

Supplement: Supplemental Information 2 — For Training purposes- Berkeley Segmentation dataset300 was used named soniya-mask. CBSD68, Set12, McMaster, and Kodak24 were used for testing purposes. [file peerj-cs-11-2449-s002.zip › overall database/CBSD68/109053.jpg]

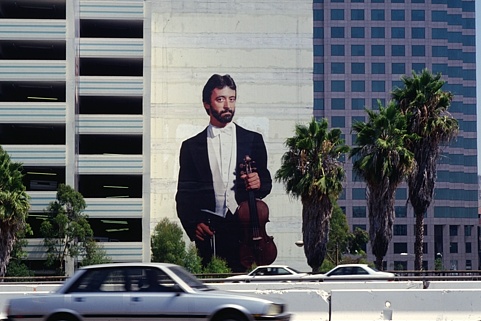

Supplement: Supplemental Information 2 — For Training purposes- Berkeley Segmentation dataset300 was used named soniya-mask. CBSD68, Set12, McMaster, and Kodak24 were used for testing purposes. [file peerj-cs-11-2449-s002.zip › overall database/CBSD68/119082.jpg]

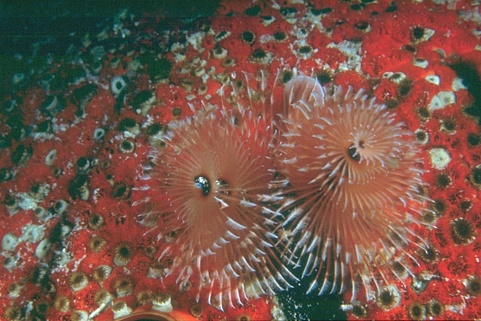

Supplement: Supplemental Information 2 — For Training purposes- Berkeley Segmentation dataset300 was used named soniya-mask. CBSD68, Set12, McMaster, and Kodak24 were used for testing purposes. [file peerj-cs-11-2449-s002.zip › overall database/CBSD68/12084.jpg]

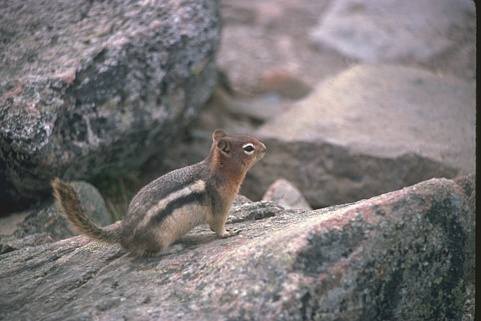

Supplement: Supplemental Information 2 — For Training purposes- Berkeley Segmentation dataset300 was used named soniya-mask. CBSD68, Set12, McMaster, and Kodak24 were used for testing purposes. [file peerj-cs-11-2449-s002.zip › overall database/CBSD68/123074.jpg]

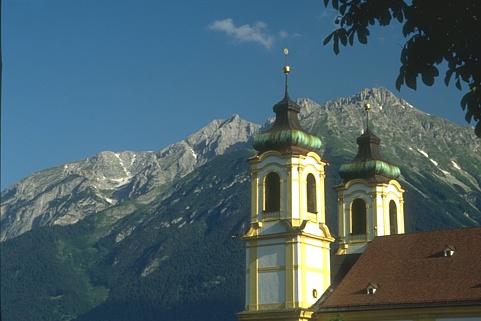

Supplement: Supplemental Information 2 — For Training purposes- Berkeley Segmentation dataset300 was used named soniya-mask. CBSD68, Set12, McMaster, and Kodak24 were used for testing purposes. [file peerj-cs-11-2449-s002.zip › overall database/CBSD68/126007.jpg]

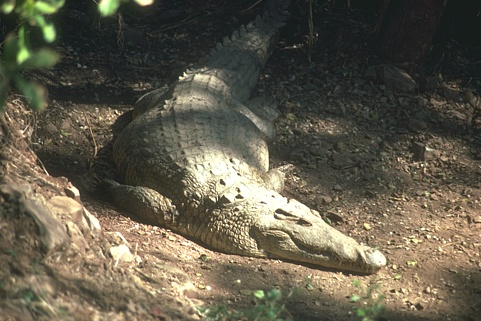

Supplement: Supplemental Information 2 — For Training purposes- Berkeley Segmentation dataset300 was used named soniya-mask. CBSD68, Set12, McMaster, and Kodak24 were used for testing purposes. [file peerj-cs-11-2449-s002.zip › overall database/CBSD68/130026.jpg]

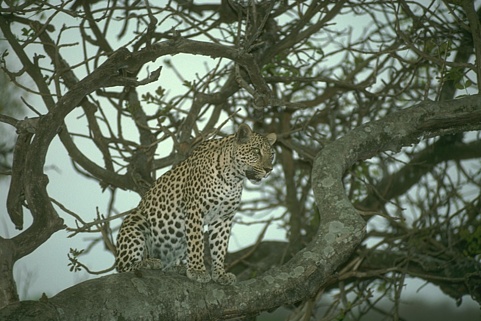

Supplement: Supplemental Information 2 — For Training purposes- Berkeley Segmentation dataset300 was used named soniya-mask. CBSD68, Set12, McMaster, and Kodak24 were used for testing purposes. [file peerj-cs-11-2449-s002.zip › overall database/CBSD68/134035.jpg]

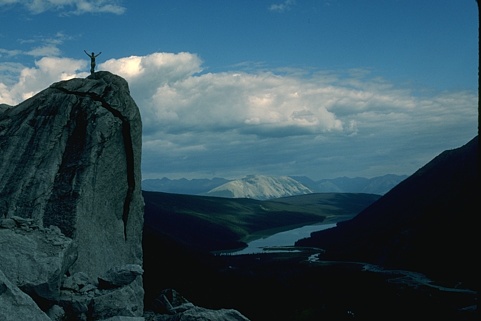

Supplement: Supplemental Information 2 — For Training purposes- Berkeley Segmentation dataset300 was used named soniya-mask. CBSD68, Set12, McMaster, and Kodak24 were used for testing purposes. [file peerj-cs-11-2449-s002.zip › overall database/CBSD68/14037.jpg]

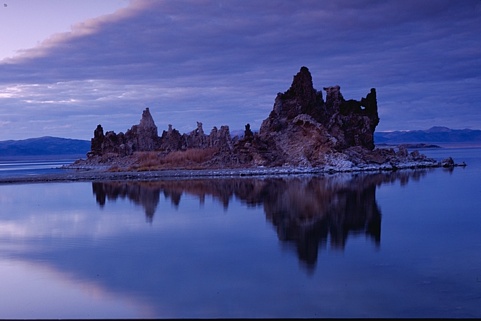

Supplement: Supplemental Information 2 — For Training purposes- Berkeley Segmentation dataset300 was used named soniya-mask. CBSD68, Set12, McMaster, and Kodak24 were used for testing purposes. [file peerj-cs-11-2449-s002.zip › overall database/CBSD68/143090.jpg]

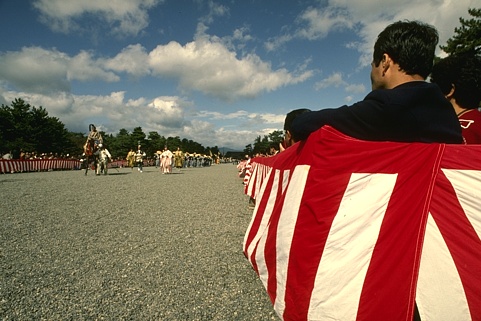

Supplement: Supplemental Information 2 — For Training purposes- Berkeley Segmentation dataset300 was used named soniya-mask. CBSD68, Set12, McMaster, and Kodak24 were used for testing purposes. [file peerj-cs-11-2449-s002.zip › overall database/CBSD68/145086.jpg]

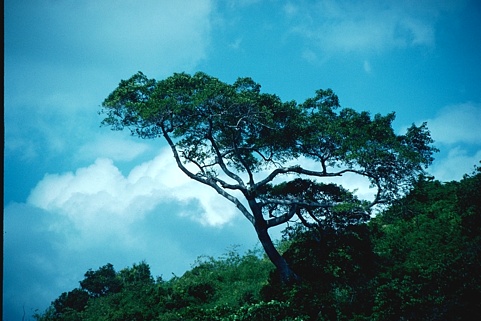

Supplement: Supplemental Information 2 — For Training purposes- Berkeley Segmentation dataset300 was used named soniya-mask. CBSD68, Set12, McMaster, and Kodak24 were used for testing purposes. [file peerj-cs-11-2449-s002.zip › overall database/CBSD68/147091.jpg]

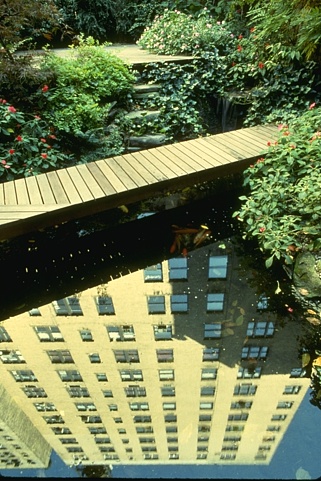

Supplement: Supplemental Information 2 — For Training purposes- Berkeley Segmentation dataset300 was used named soniya-mask. CBSD68, Set12, McMaster, and Kodak24 were used for testing purposes. [file peerj-cs-11-2449-s002.zip › overall database/CBSD68/148026.jpg]

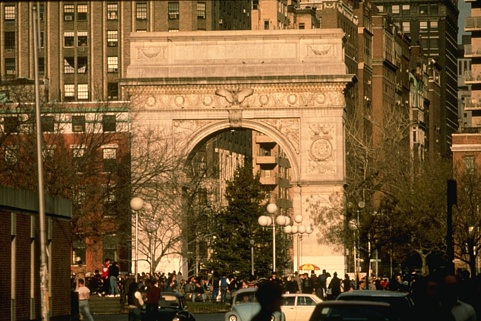

Supplement: Supplemental Information 2 — For Training purposes- Berkeley Segmentation dataset300 was used named soniya-mask. CBSD68, Set12, McMaster, and Kodak24 were used for testing purposes. [file peerj-cs-11-2449-s002.zip › overall database/CBSD68/148089.jpg]

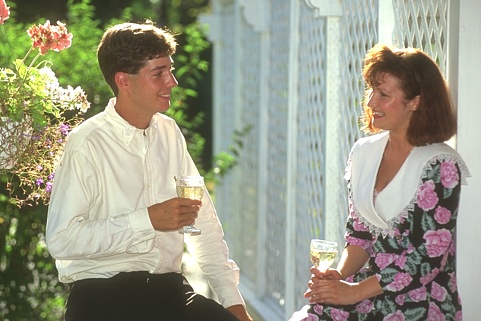

Supplement: Supplemental Information 2 — For Training purposes- Berkeley Segmentation dataset300 was used named soniya-mask. CBSD68, Set12, McMaster, and Kodak24 were used for testing purposes. [file peerj-cs-11-2449-s002.zip › overall database/CBSD68/157055.jpg]

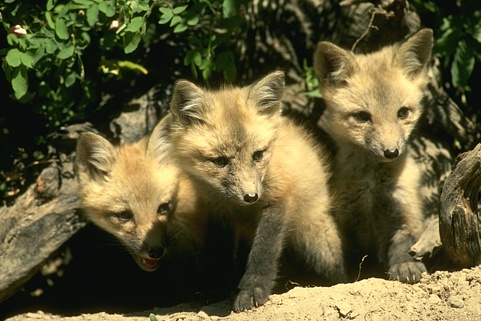

Supplement: Supplemental Information 2 — For Training purposes- Berkeley Segmentation dataset300 was used named soniya-mask. CBSD68, Set12, McMaster, and Kodak24 were used for testing purposes. [file peerj-cs-11-2449-s002.zip › overall database/CBSD68/159008.jpg]

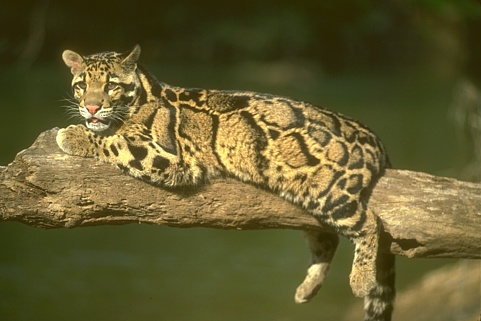

Supplement: Supplemental Information 2 — For Training purposes- Berkeley Segmentation dataset300 was used named soniya-mask. CBSD68, Set12, McMaster, and Kodak24 were used for testing purposes. [file peerj-cs-11-2449-s002.zip › overall database/CBSD68/160068.jpg]

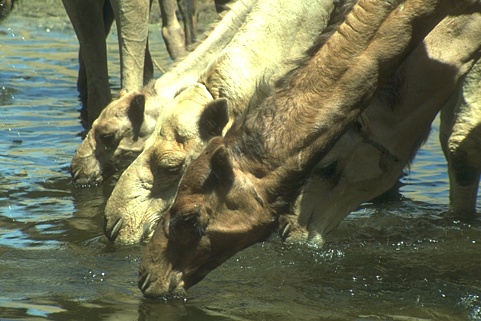

Supplement: Supplemental Information 2 — For Training purposes- Berkeley Segmentation dataset300 was used named soniya-mask. CBSD68, Set12, McMaster, and Kodak24 were used for testing purposes. [file peerj-cs-11-2449-s002.zip › overall database/CBSD68/16077.jpg]

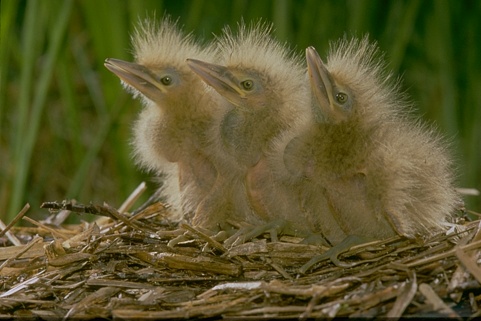

Supplement: Supplemental Information 2 — For Training purposes- Berkeley Segmentation dataset300 was used named soniya-mask. CBSD68, Set12, McMaster, and Kodak24 were used for testing purposes. [file peerj-cs-11-2449-s002.zip › overall database/CBSD68/163085.jpg]

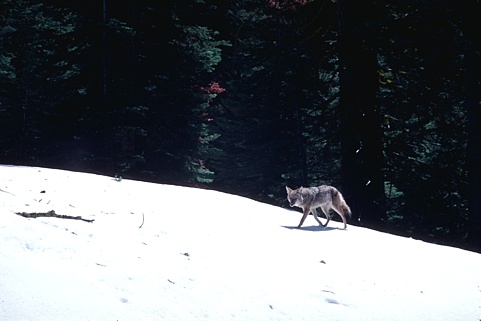

Supplement: Supplemental Information 2 — For Training purposes- Berkeley Segmentation dataset300 was used named soniya-mask. CBSD68, Set12, McMaster, and Kodak24 were used for testing purposes. [file peerj-cs-11-2449-s002.zip › overall database/CBSD68/167062.jpg]

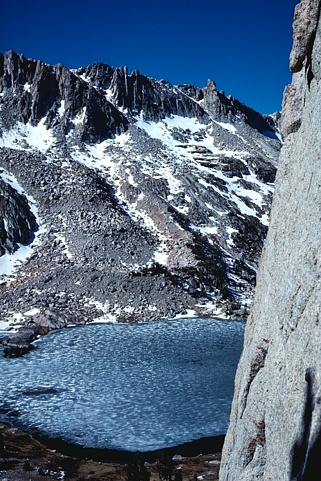

Supplement: Supplemental Information 2 — For Training purposes- Berkeley Segmentation dataset300 was used named soniya-mask. CBSD68, Set12, McMaster, and Kodak24 were used for testing purposes. [file peerj-cs-11-2449-s002.zip › overall database/CBSD68/167083.jpg]

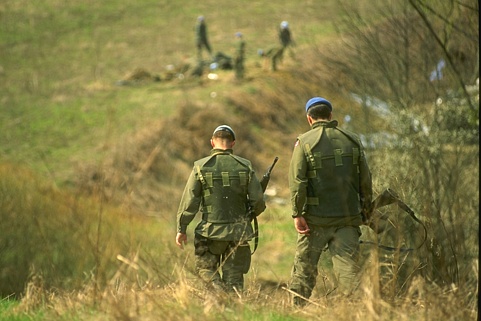

Supplement: Supplemental Information 2 — For Training purposes- Berkeley Segmentation dataset300 was used named soniya-mask. CBSD68, Set12, McMaster, and Kodak24 were used for testing purposes. [file peerj-cs-11-2449-s002.zip › overall database/CBSD68/170057.jpg]

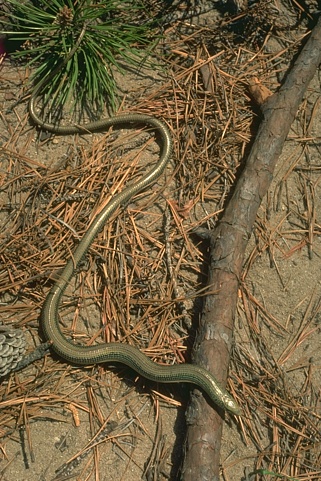

Supplement: Supplemental Information 2 — For Training purposes- Berkeley Segmentation dataset300 was used named soniya-mask. CBSD68, Set12, McMaster, and Kodak24 were used for testing purposes. [file peerj-cs-11-2449-s002.zip › overall database/CBSD68/175032.jpg]

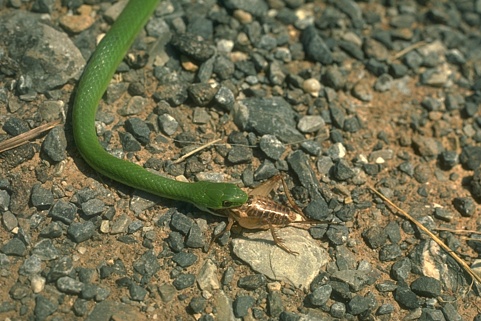

Supplement: Supplemental Information 2 — For Training purposes- Berkeley Segmentation dataset300 was used named soniya-mask. CBSD68, Set12, McMaster, and Kodak24 were used for testing purposes. [file peerj-cs-11-2449-s002.zip › overall database/CBSD68/175043.jpg]

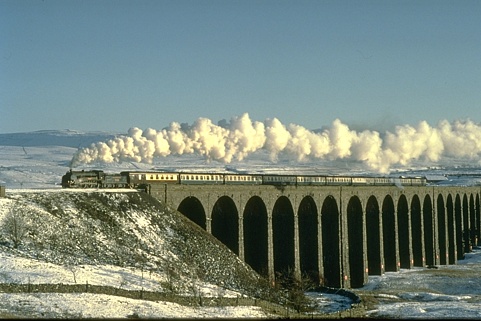

Supplement: Supplemental Information 2 — For Training purposes- Berkeley Segmentation dataset300 was used named soniya-mask. CBSD68, Set12, McMaster, and Kodak24 were used for testing purposes. [file peerj-cs-11-2449-s002.zip › overall database/CBSD68/182053.jpg]

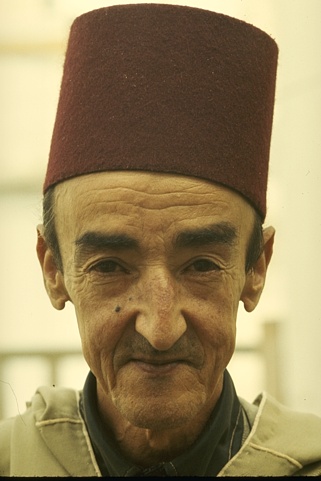

Supplement: Supplemental Information 2 — For Training purposes- Berkeley Segmentation dataset300 was used named soniya-mask. CBSD68, Set12, McMaster, and Kodak24 were used for testing purposes. [file peerj-cs-11-2449-s002.zip › overall database/CBSD68/189080.jpg]

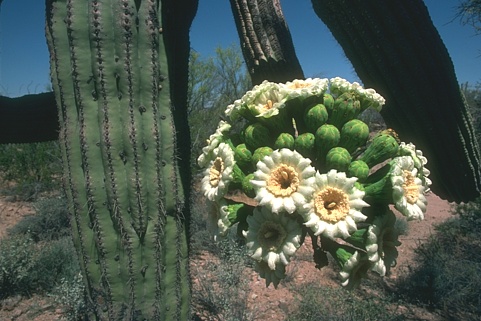

Supplement: Supplemental Information 2 — For Training purposes- Berkeley Segmentation dataset300 was used named soniya-mask. CBSD68, Set12, McMaster, and Kodak24 were used for testing purposes. [file peerj-cs-11-2449-s002.zip › overall database/CBSD68/19021.jpg]

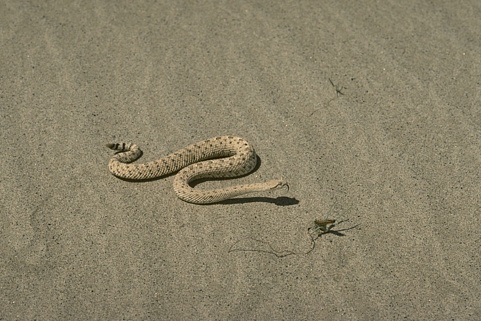

Supplement: Supplemental Information 2 — For Training purposes- Berkeley Segmentation dataset300 was used named soniya-mask. CBSD68, Set12, McMaster, and Kodak24 were used for testing purposes. [file peerj-cs-11-2449-s002.zip › overall database/CBSD68/196073.jpg]

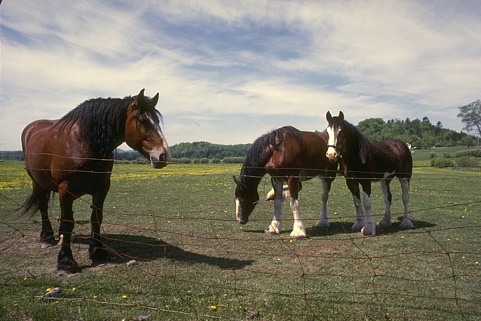

Supplement: Supplemental Information 2 — For Training purposes- Berkeley Segmentation dataset300 was used named soniya-mask. CBSD68, Set12, McMaster, and Kodak24 were used for testing purposes. [file peerj-cs-11-2449-s002.zip › overall database/CBSD68/197017.jpg]

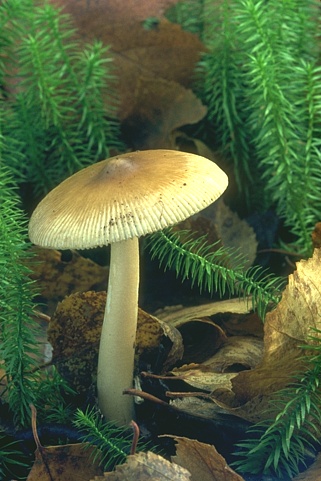

Supplement: Supplemental Information 2 — For Training purposes- Berkeley Segmentation dataset300 was used named soniya-mask. CBSD68, Set12, McMaster, and Kodak24 were used for testing purposes. [file peerj-cs-11-2449-s002.zip › overall database/CBSD68/208001.jpg]

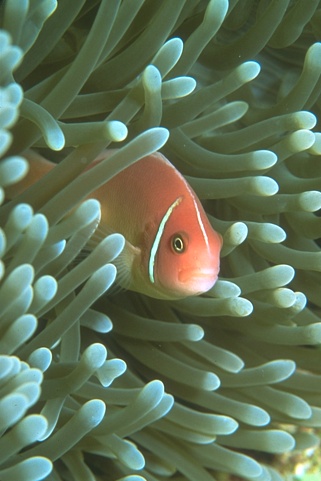

Supplement: Supplemental Information 2 — For Training purposes- Berkeley Segmentation dataset300 was used named soniya-mask. CBSD68, Set12, McMaster, and Kodak24 were used for testing purposes. [file peerj-cs-11-2449-s002.zip › overall database/CBSD68/210088.jpg]

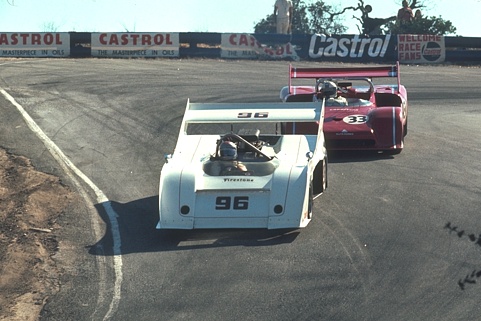

Supplement: Supplemental Information 2 — For Training purposes- Berkeley Segmentation dataset300 was used named soniya-mask. CBSD68, Set12, McMaster, and Kodak24 were used for testing purposes. [file peerj-cs-11-2449-s002.zip › overall database/CBSD68/21077.jpg]

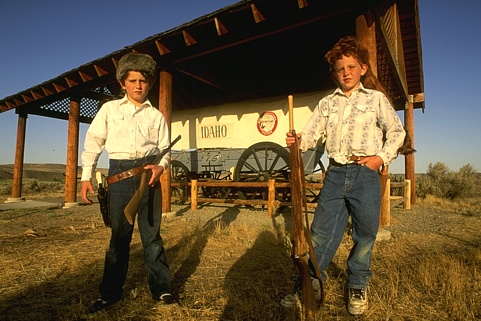

Supplement: Supplemental Information 2 — For Training purposes- Berkeley Segmentation dataset300 was used named soniya-mask. CBSD68, Set12, McMaster, and Kodak24 were used for testing purposes. [file peerj-cs-11-2449-s002.zip › overall database/CBSD68/216081.jpg]

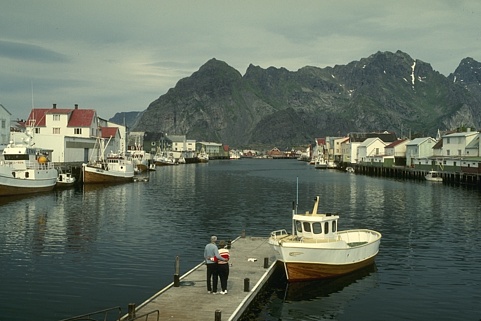

Supplement: Supplemental Information 2 — For Training purposes- Berkeley Segmentation dataset300 was used named soniya-mask. CBSD68, Set12, McMaster, and Kodak24 were used for testing purposes. [file peerj-cs-11-2449-s002.zip › overall database/CBSD68/219090.jpg]

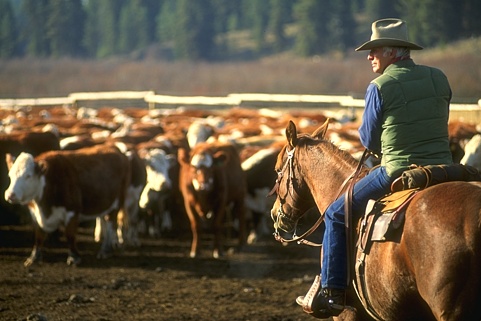

Supplement: Supplemental Information 2 — For Training purposes- Berkeley Segmentation dataset300 was used named soniya-mask. CBSD68, Set12, McMaster, and Kodak24 were used for testing purposes. [file peerj-cs-11-2449-s002.zip › overall database/CBSD68/220075.jpg]

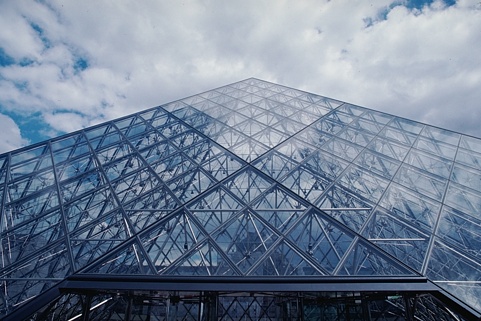

Supplement: Supplemental Information 2 — For Training purposes- Berkeley Segmentation dataset300 was used named soniya-mask. CBSD68, Set12, McMaster, and Kodak24 were used for testing purposes. [file peerj-cs-11-2449-s002.zip › overall database/CBSD68/223061.jpg]

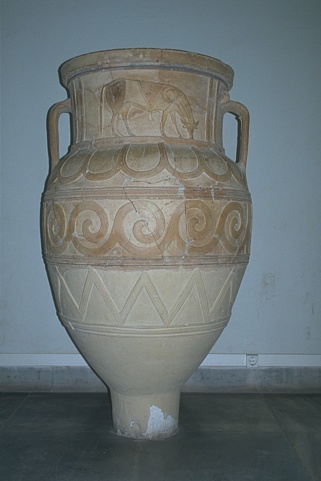

Supplement: Supplemental Information 2 — For Training purposes- Berkeley Segmentation dataset300 was used named soniya-mask. CBSD68, Set12, McMaster, and Kodak24 were used for testing purposes. [file peerj-cs-11-2449-s002.zip › overall database/CBSD68/227092.jpg]

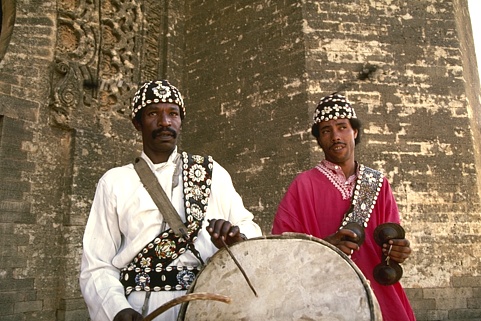

Supplement: Supplemental Information 2 — For Training purposes- Berkeley Segmentation dataset300 was used named soniya-mask. CBSD68, Set12, McMaster, and Kodak24 were used for testing purposes. [file peerj-cs-11-2449-s002.zip › overall database/CBSD68/229036.jpg]

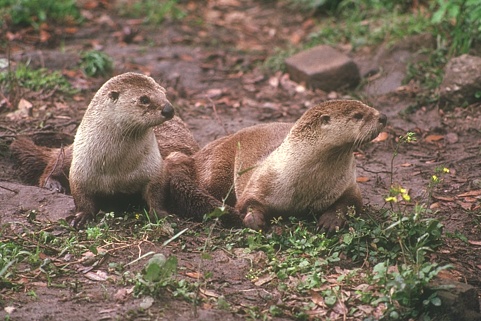

Supplement: Supplemental Information 2 — For Training purposes- Berkeley Segmentation dataset300 was used named soniya-mask. CBSD68, Set12, McMaster, and Kodak24 were used for testing purposes. [file peerj-cs-11-2449-s002.zip › overall database/CBSD68/236037.jpg]

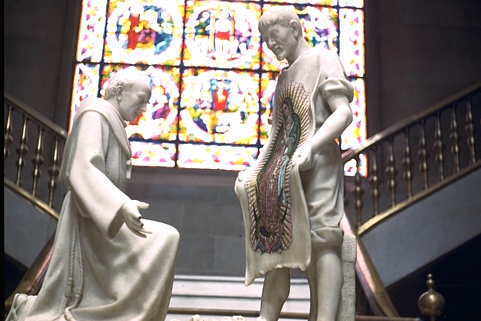

Supplement: Supplemental Information 2 — For Training purposes- Berkeley Segmentation dataset300 was used named soniya-mask. CBSD68, Set12, McMaster, and Kodak24 were used for testing purposes. [file peerj-cs-11-2449-s002.zip › overall database/CBSD68/24077.jpg]

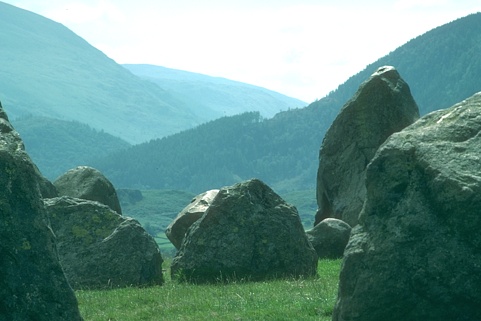

Supplement: Supplemental Information 2 — For Training purposes- Berkeley Segmentation dataset300 was used named soniya-mask. CBSD68, Set12, McMaster, and Kodak24 were used for testing purposes. [file peerj-cs-11-2449-s002.zip › overall database/CBSD68/241004.jpg]

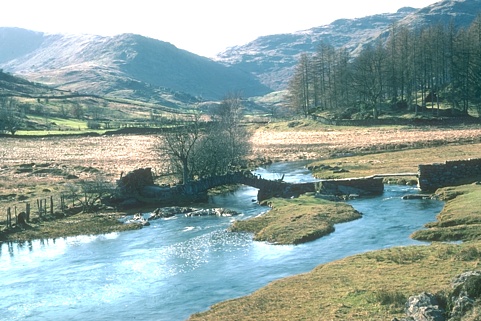

Supplement: Supplemental Information 2 — For Training purposes- Berkeley Segmentation dataset300 was used named soniya-mask. CBSD68, Set12, McMaster, and Kodak24 were used for testing purposes. [file peerj-cs-11-2449-s002.zip › overall database/CBSD68/241048.jpg]

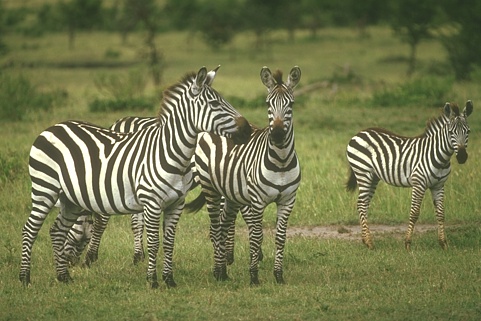

Supplement: Supplemental Information 2 — For Training purposes- Berkeley Segmentation dataset300 was used named soniya-mask. CBSD68, Set12, McMaster, and Kodak24 were used for testing purposes. [file peerj-cs-11-2449-s002.zip › overall database/CBSD68/253027.jpg]

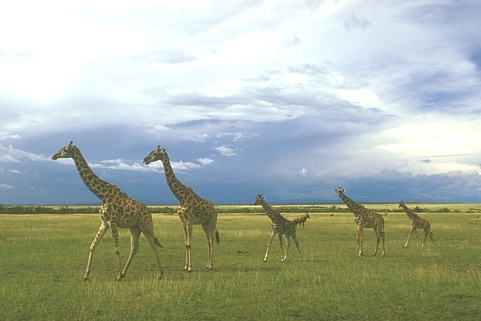

Supplement: Supplemental Information 2 — For Training purposes- Berkeley Segmentation dataset300 was used named soniya-mask. CBSD68, Set12, McMaster, and Kodak24 were used for testing purposes. [file peerj-cs-11-2449-s002.zip › overall database/CBSD68/253055.jpg]

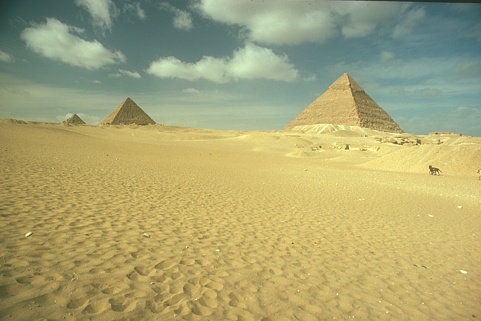

Supplement: Supplemental Information 2 — For Training purposes- Berkeley Segmentation dataset300 was used named soniya-mask. CBSD68, Set12, McMaster, and Kodak24 were used for testing purposes. [file peerj-cs-11-2449-s002.zip › overall database/CBSD68/260058.jpg]

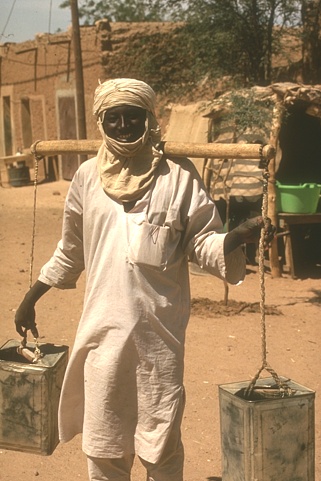

Supplement: Supplemental Information 2 — For Training purposes- Berkeley Segmentation dataset300 was used named soniya-mask. CBSD68, Set12, McMaster, and Kodak24 were used for testing purposes. [file peerj-cs-11-2449-s002.zip › overall database/CBSD68/271035.jpg]

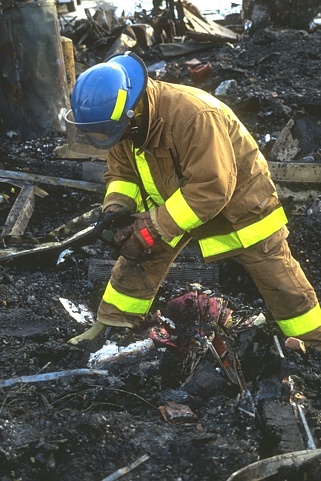

Supplement: Supplemental Information 2 — For Training purposes- Berkeley Segmentation dataset300 was used named soniya-mask. CBSD68, Set12, McMaster, and Kodak24 were used for testing purposes. [file peerj-cs-11-2449-s002.zip › overall database/CBSD68/285079.jpg]

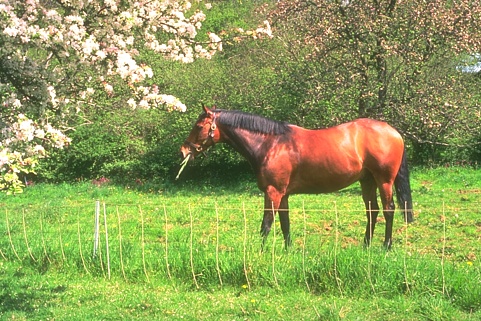

Supplement: Supplemental Information 2 — For Training purposes- Berkeley Segmentation dataset300 was used named soniya-mask. CBSD68, Set12, McMaster, and Kodak24 were used for testing purposes. [file peerj-cs-11-2449-s002.zip › overall database/CBSD68/291000.jpg]

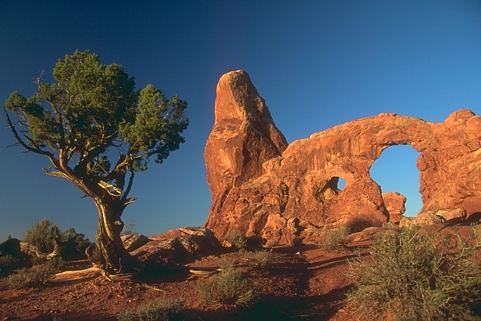

Supplement: Supplemental Information 2 — For Training purposes- Berkeley Segmentation dataset300 was used named soniya-mask. CBSD68, Set12, McMaster, and Kodak24 were used for testing purposes. [file peerj-cs-11-2449-s002.zip › overall database/CBSD68/295087.jpg]

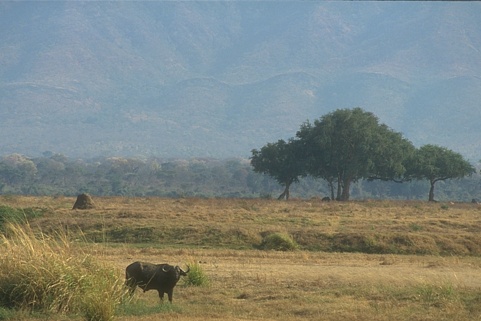

Supplement: Supplemental Information 2 — For Training purposes- Berkeley Segmentation dataset300 was used named soniya-mask. CBSD68, Set12, McMaster, and Kodak24 were used for testing purposes. [file peerj-cs-11-2449-s002.zip › overall database/CBSD68/296007.jpg]

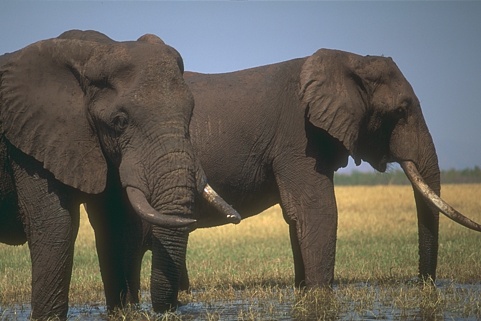

Supplement: Supplemental Information 2 — For Training purposes- Berkeley Segmentation dataset300 was used named soniya-mask. CBSD68, Set12, McMaster, and Kodak24 were used for testing purposes. [file peerj-cs-11-2449-s002.zip › overall database/CBSD68/296059.jpg]

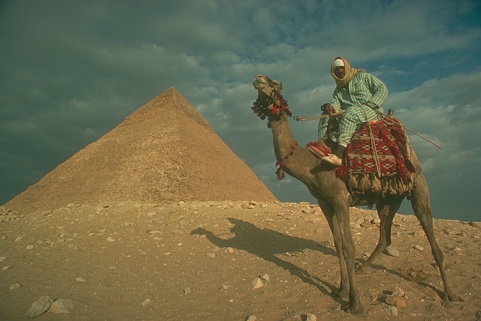

Supplement: Supplemental Information 2 — For Training purposes- Berkeley Segmentation dataset300 was used named soniya-mask. CBSD68, Set12, McMaster, and Kodak24 were used for testing purposes. [file peerj-cs-11-2449-s002.zip › overall database/CBSD68/299086.jpg]

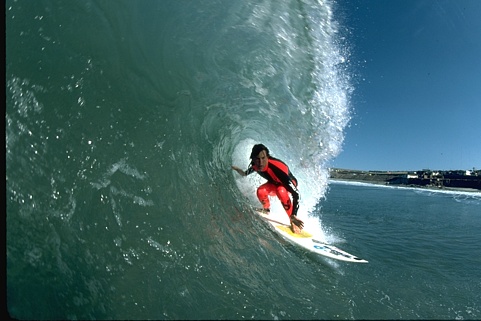

Supplement: Supplemental Information 2 — For Training purposes- Berkeley Segmentation dataset300 was used named soniya-mask. CBSD68, Set12, McMaster, and Kodak24 were used for testing purposes. [file peerj-cs-11-2449-s002.zip › overall database/CBSD68/300091.jpg]

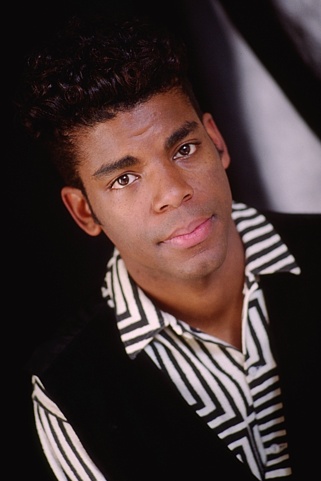

Supplement: Supplemental Information 2 — For Training purposes- Berkeley Segmentation dataset300 was used named soniya-mask. CBSD68, Set12, McMaster, and Kodak24 were used for testing purposes. [file peerj-cs-11-2449-s002.zip › overall database/CBSD68/302008.jpg]

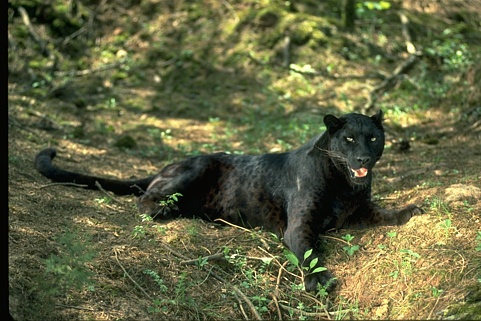

Supplement: Supplemental Information 2 — For Training purposes- Berkeley Segmentation dataset300 was used named soniya-mask. CBSD68, Set12, McMaster, and Kodak24 were used for testing purposes. [file peerj-cs-11-2449-s002.zip › overall database/CBSD68/304034.jpg]

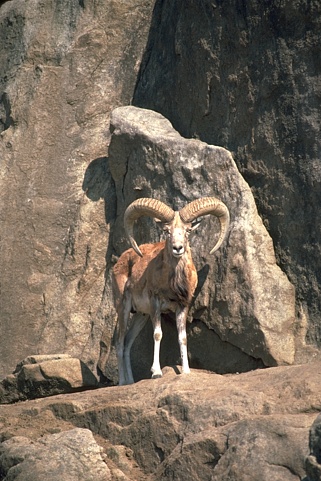

Supplement: Supplemental Information 2 — For Training purposes- Berkeley Segmentation dataset300 was used named soniya-mask. CBSD68, Set12, McMaster, and Kodak24 were used for testing purposes. [file peerj-cs-11-2449-s002.zip › overall database/CBSD68/304074.jpg]

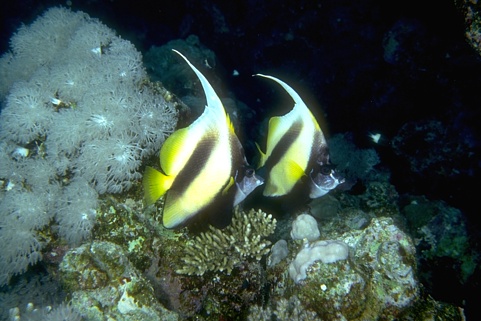

Supplement: Supplemental Information 2 — For Training purposes- Berkeley Segmentation dataset300 was used named soniya-mask. CBSD68, Set12, McMaster, and Kodak24 were used for testing purposes. [file peerj-cs-11-2449-s002.zip › overall database/CBSD68/306005.jpg]

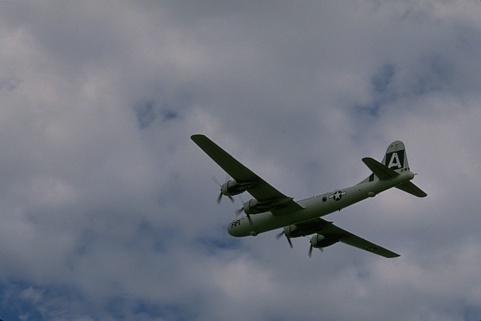

Supplement: Supplemental Information 2 — For Training purposes- Berkeley Segmentation dataset300 was used named soniya-mask. CBSD68, Set12, McMaster, and Kodak24 were used for testing purposes. [file peerj-cs-11-2449-s002.zip › overall database/CBSD68/3096.jpg]

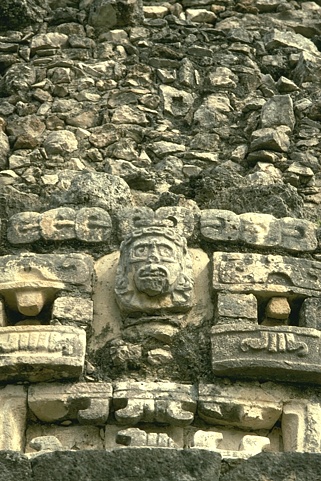

Supplement: Supplemental Information 2 — For Training purposes- Berkeley Segmentation dataset300 was used named soniya-mask. CBSD68, Set12, McMaster, and Kodak24 were used for testing purposes. [file peerj-cs-11-2449-s002.zip › overall database/CBSD68/33039.jpg]

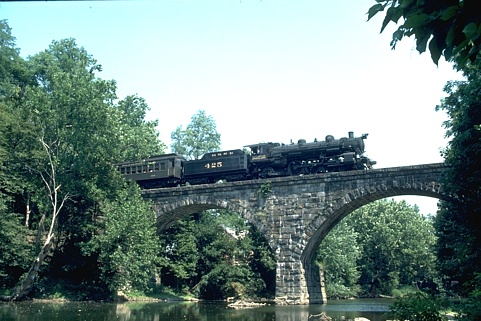

Supplement: Supplemental Information 2 — For Training purposes- Berkeley Segmentation dataset300 was used named soniya-mask. CBSD68, Set12, McMaster, and Kodak24 were used for testing purposes. [file peerj-cs-11-2449-s002.zip › overall database/CBSD68/351093.jpg]

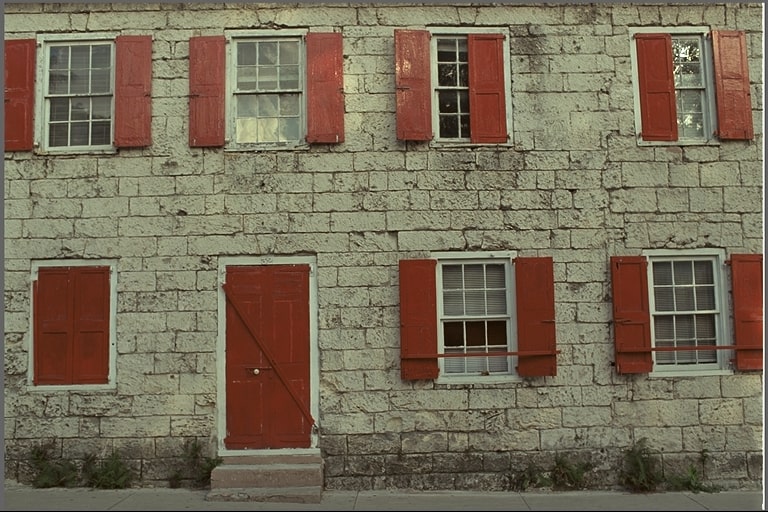

Supplement: Supplemental Information 2 — For Training purposes- Berkeley Segmentation dataset300 was used named soniya-mask. CBSD68, Set12, McMaster, and Kodak24 were used for testing purposes. [file peerj-cs-11-2449-s002.zip › overall database/kodak24/kodim01.jpg]

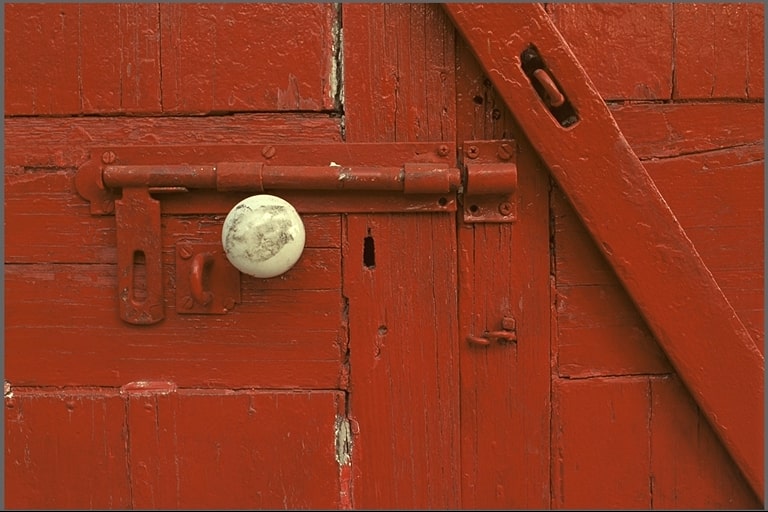

Supplement: Supplemental Information 2 — For Training purposes- Berkeley Segmentation dataset300 was used named soniya-mask. CBSD68, Set12, McMaster, and Kodak24 were used for testing purposes. [file peerj-cs-11-2449-s002.zip › overall database/kodak24/kodim02.jpg]

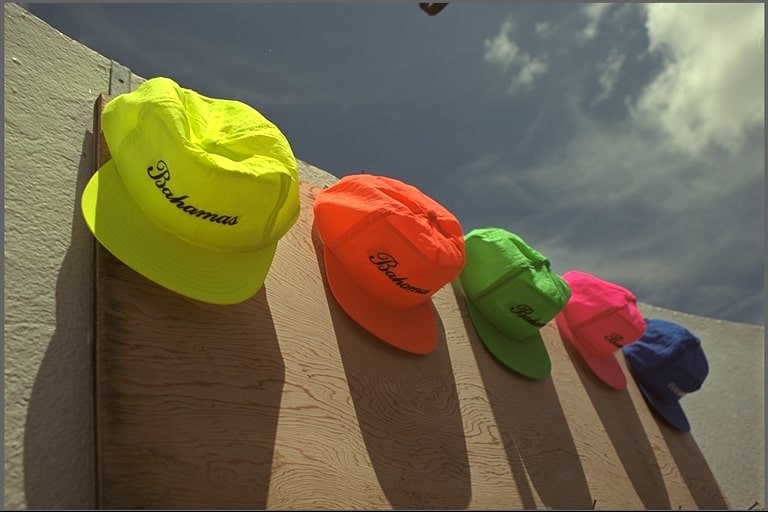

Supplement: Supplemental Information 2 — For Training purposes- Berkeley Segmentation dataset300 was used named soniya-mask. CBSD68, Set12, McMaster, and Kodak24 were used for testing purposes. [file peerj-cs-11-2449-s002.zip › overall database/kodak24/kodim03.jpg]

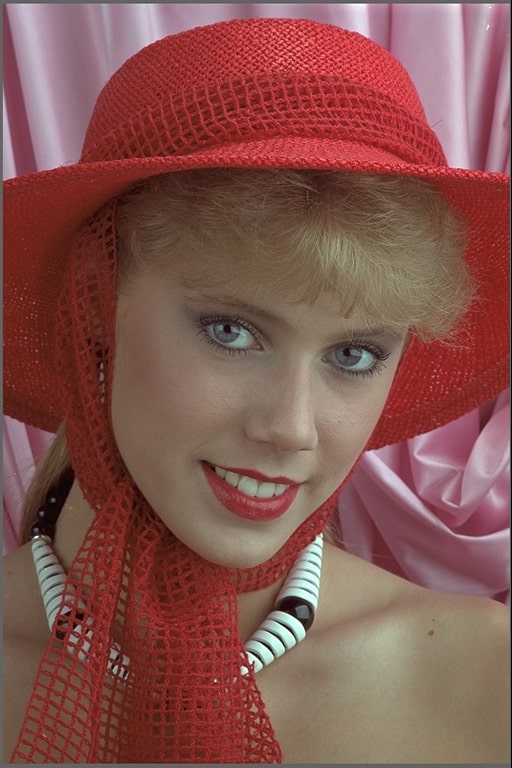

Supplement: Supplemental Information 2 — For Training purposes- Berkeley Segmentation dataset300 was used named soniya-mask. CBSD68, Set12, McMaster, and Kodak24 were used for testing purposes. [file peerj-cs-11-2449-s002.zip › overall database/kodak24/kodim04.jpg]

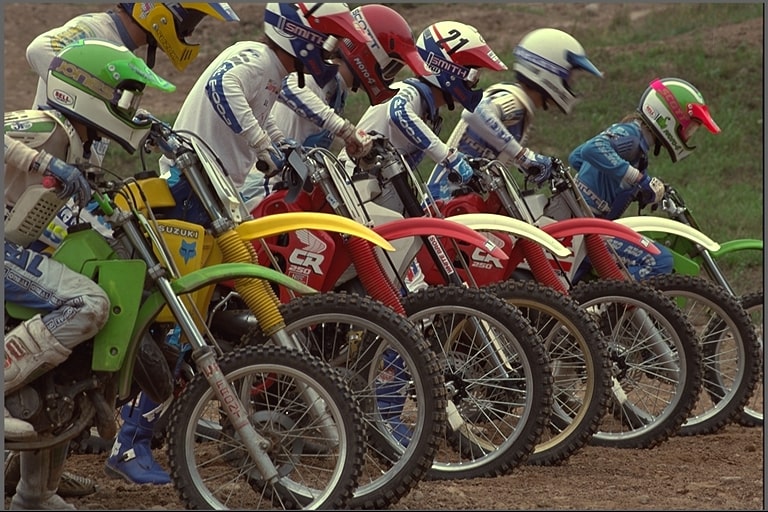

Supplement: Supplemental Information 2 — For Training purposes- Berkeley Segmentation dataset300 was used named soniya-mask. CBSD68, Set12, McMaster, and Kodak24 were used for testing purposes. [file peerj-cs-11-2449-s002.zip › overall database/kodak24/kodim05.jpg]

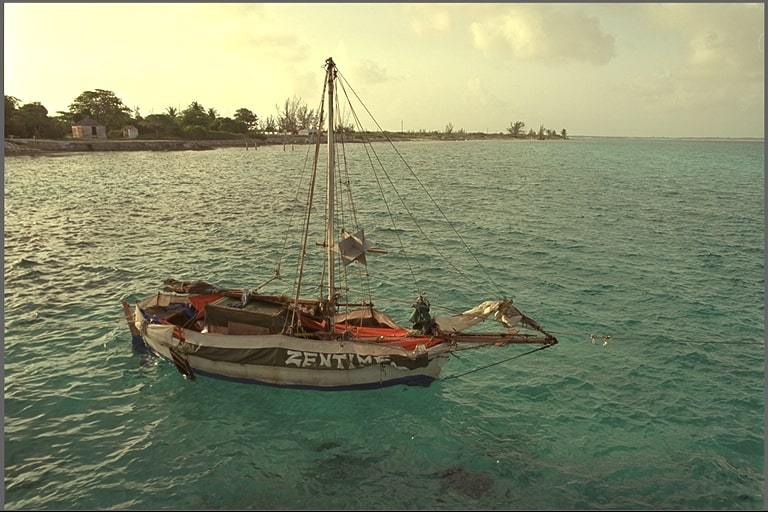

Supplement: Supplemental Information 2 — For Training purposes- Berkeley Segmentation dataset300 was used named soniya-mask. CBSD68, Set12, McMaster, and Kodak24 were used for testing purposes. [file peerj-cs-11-2449-s002.zip › overall database/kodak24/kodim06.jpg]

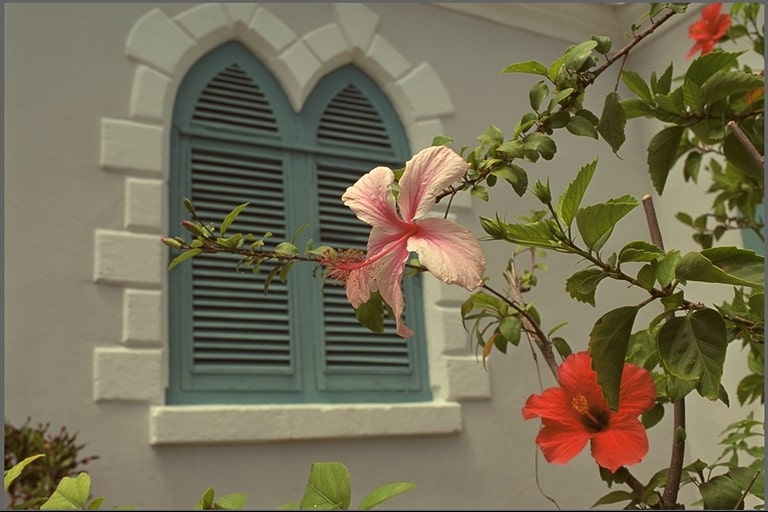

Supplement: Supplemental Information 2 — For Training purposes- Berkeley Segmentation dataset300 was used named soniya-mask. CBSD68, Set12, McMaster, and Kodak24 were used for testing purposes. [file peerj-cs-11-2449-s002.zip › overall database/kodak24/kodim07.jpg]

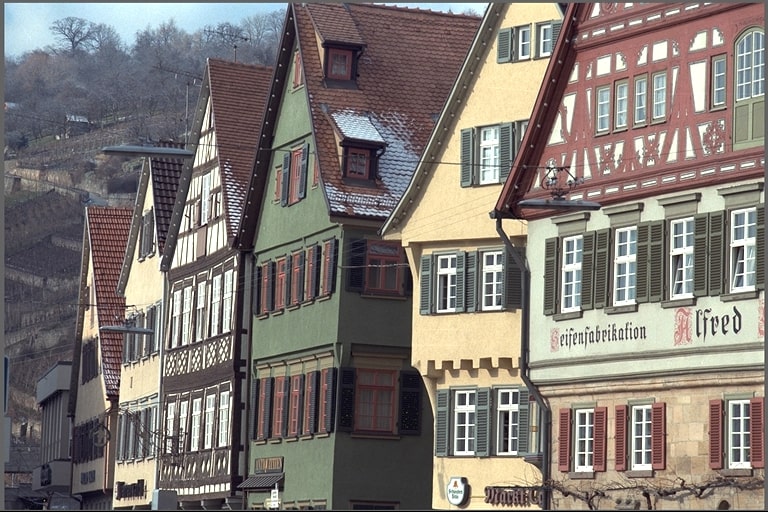

Supplement: Supplemental Information 2 — For Training purposes- Berkeley Segmentation dataset300 was used named soniya-mask. CBSD68, Set12, McMaster, and Kodak24 were used for testing purposes. [file peerj-cs-11-2449-s002.zip › overall database/kodak24/kodim08.jpg]

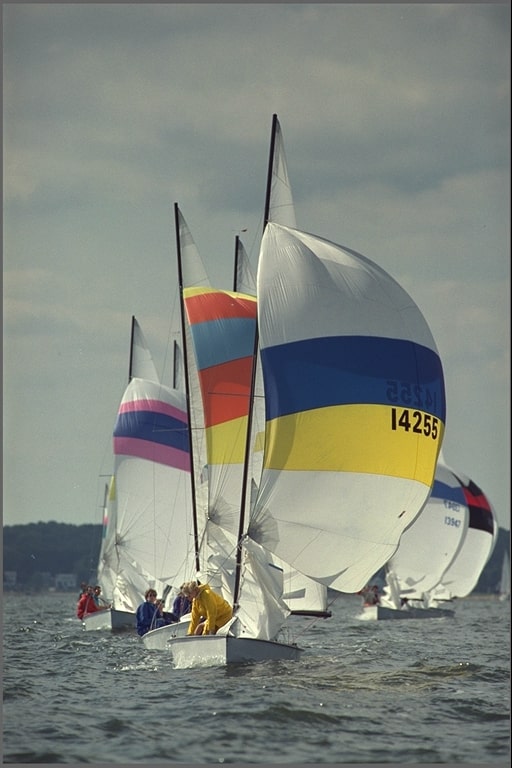

Supplement: Supplemental Information 2 — For Training purposes- Berkeley Segmentation dataset300 was used named soniya-mask. CBSD68, Set12, McMaster, and Kodak24 were used for testing purposes. [file peerj-cs-11-2449-s002.zip › overall database/kodak24/kodim09.jpg]

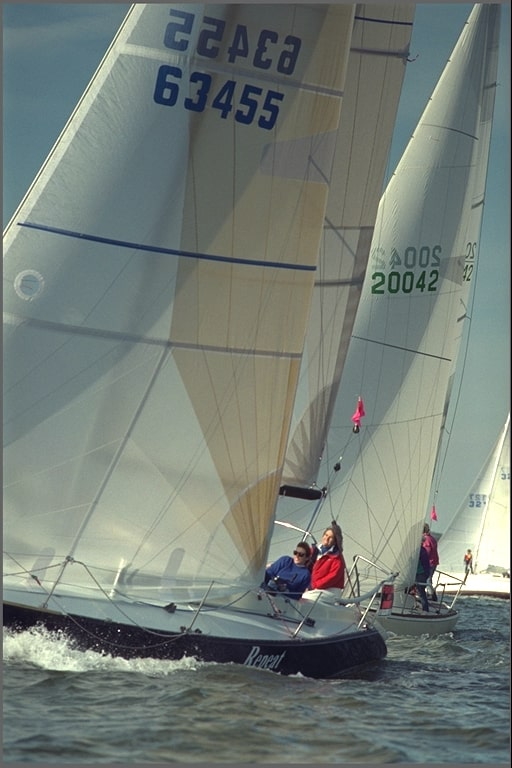

Supplement: Supplemental Information 2 — For Training purposes- Berkeley Segmentation dataset300 was used named soniya-mask. CBSD68, Set12, McMaster, and Kodak24 were used for testing purposes. [file peerj-cs-11-2449-s002.zip › overall database/kodak24/kodim10.jpg]

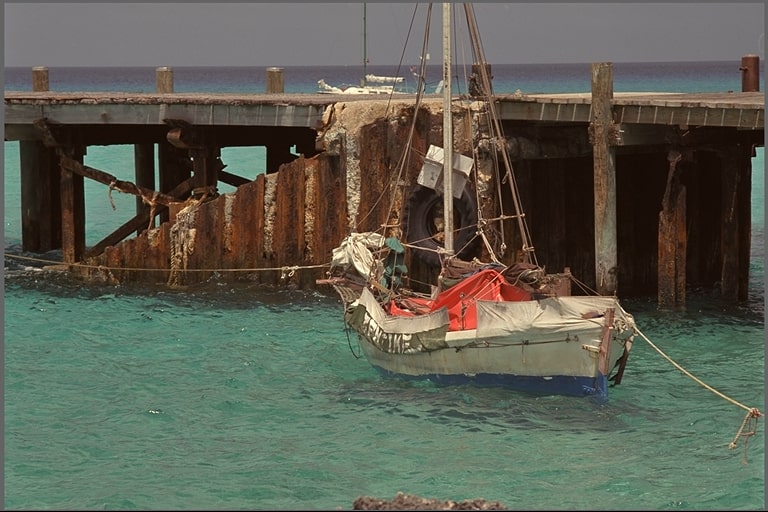

Supplement: Supplemental Information 2 — For Training purposes- Berkeley Segmentation dataset300 was used named soniya-mask. CBSD68, Set12, McMaster, and Kodak24 were used for testing purposes. [file peerj-cs-11-2449-s002.zip › overall database/kodak24/kodim11.jpg]

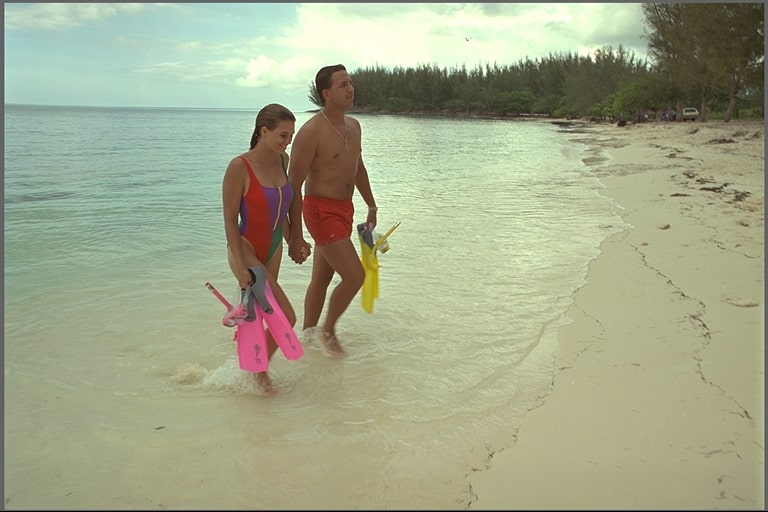

Supplement: Supplemental Information 2 — For Training purposes- Berkeley Segmentation dataset300 was used named soniya-mask. CBSD68, Set12, McMaster, and Kodak24 were used for testing purposes. [file peerj-cs-11-2449-s002.zip › overall database/kodak24/kodim12.jpg]

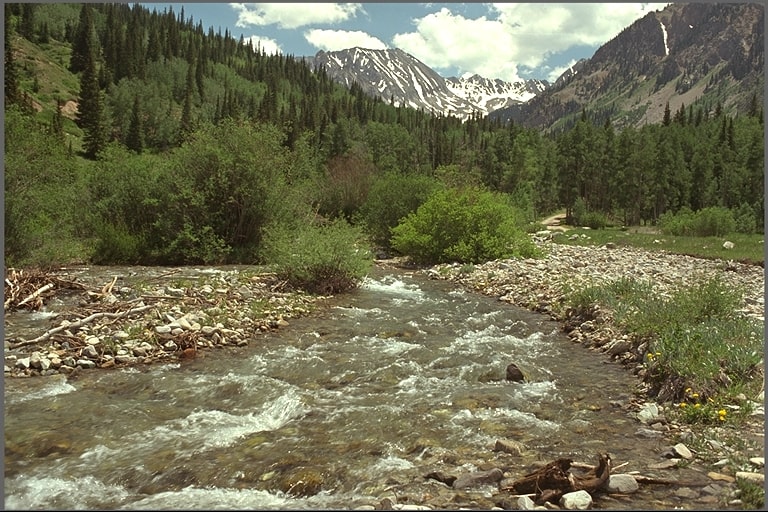

Supplement: Supplemental Information 2 — For Training purposes- Berkeley Segmentation dataset300 was used named soniya-mask. CBSD68, Set12, McMaster, and Kodak24 were used for testing purposes. [file peerj-cs-11-2449-s002.zip › overall database/kodak24/kodim13.jpg]

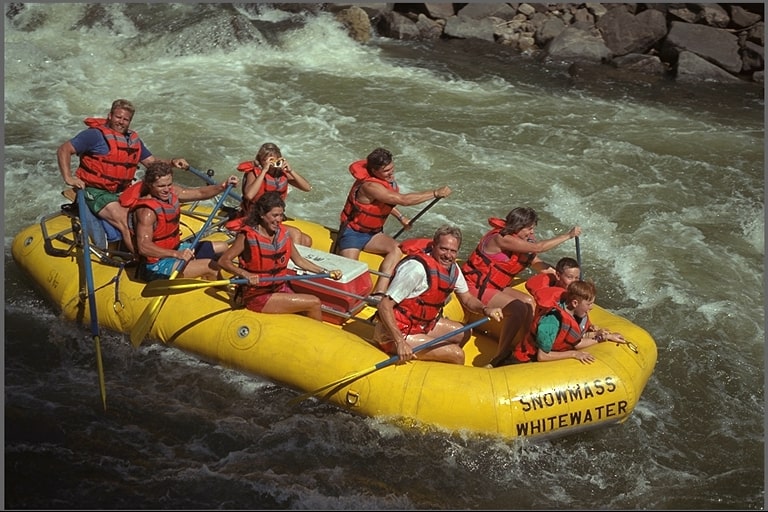

Supplement: Supplemental Information 2 — For Training purposes- Berkeley Segmentation dataset300 was used named soniya-mask. CBSD68, Set12, McMaster, and Kodak24 were used for testing purposes. [file peerj-cs-11-2449-s002.zip › overall database/kodak24/kodim14.jpg]

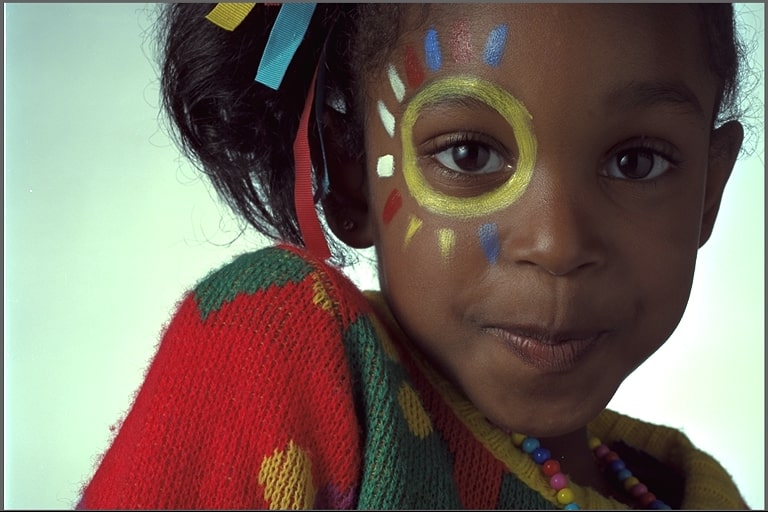

Supplement: Supplemental Information 2 — For Training purposes- Berkeley Segmentation dataset300 was used named soniya-mask. CBSD68, Set12, McMaster, and Kodak24 were used for testing purposes. [file peerj-cs-11-2449-s002.zip › overall database/kodak24/kodim15.jpg]

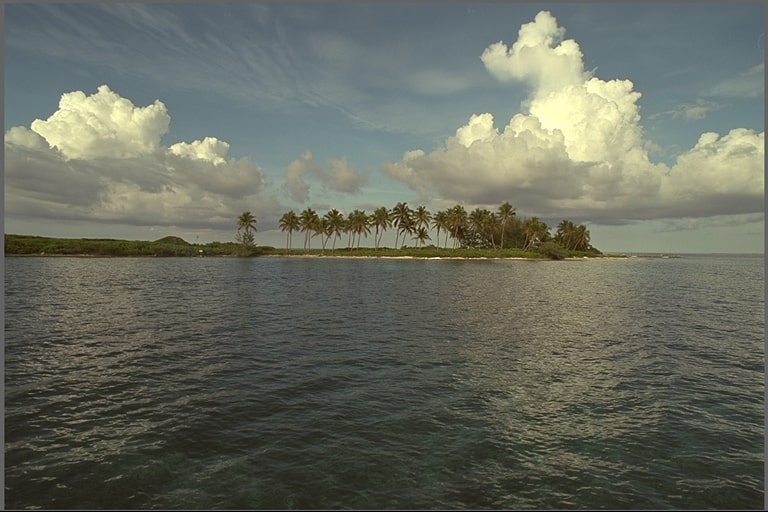

Supplement: Supplemental Information 2 — For Training purposes- Berkeley Segmentation dataset300 was used named soniya-mask. CBSD68, Set12, McMaster, and Kodak24 were used for testing purposes. [file peerj-cs-11-2449-s002.zip › overall database/kodak24/kodim16.jpg]

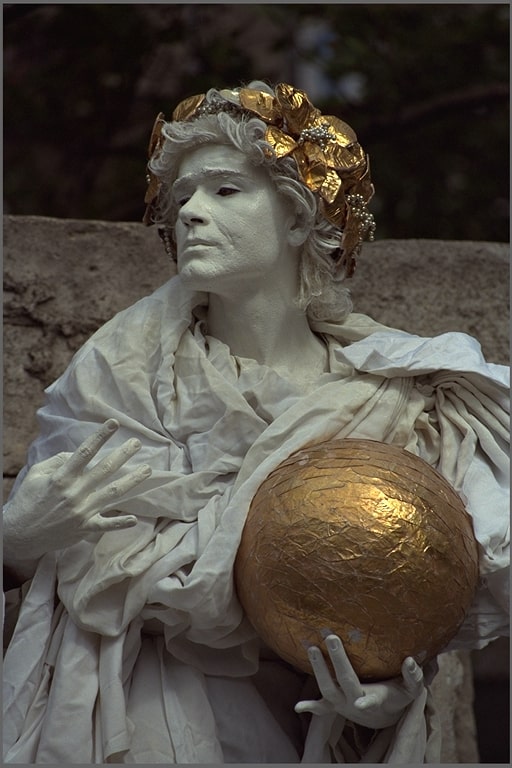

Supplement: Supplemental Information 2 — For Training purposes- Berkeley Segmentation dataset300 was used named soniya-mask. CBSD68, Set12, McMaster, and Kodak24 were used for testing purposes. [file peerj-cs-11-2449-s002.zip › overall database/kodak24/kodim17.jpg]

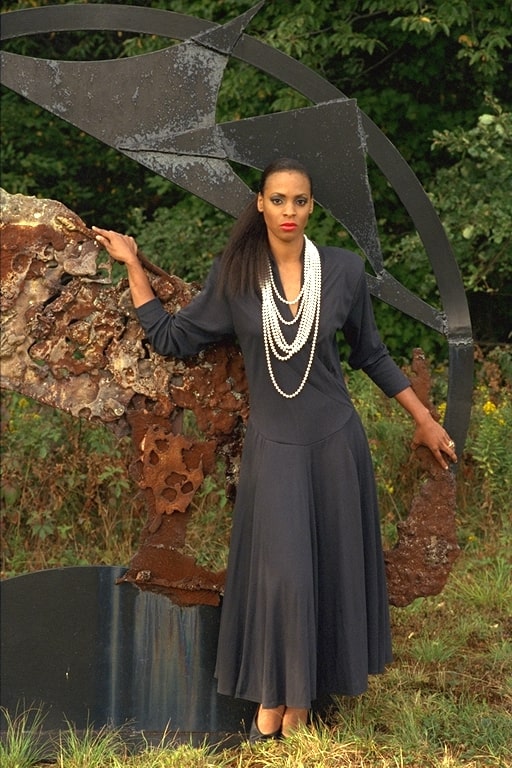

Supplement: Supplemental Information 2 — For Training purposes- Berkeley Segmentation dataset300 was used named soniya-mask. CBSD68, Set12, McMaster, and Kodak24 were used for testing purposes. [file peerj-cs-11-2449-s002.zip › overall database/kodak24/kodim18.jpg]

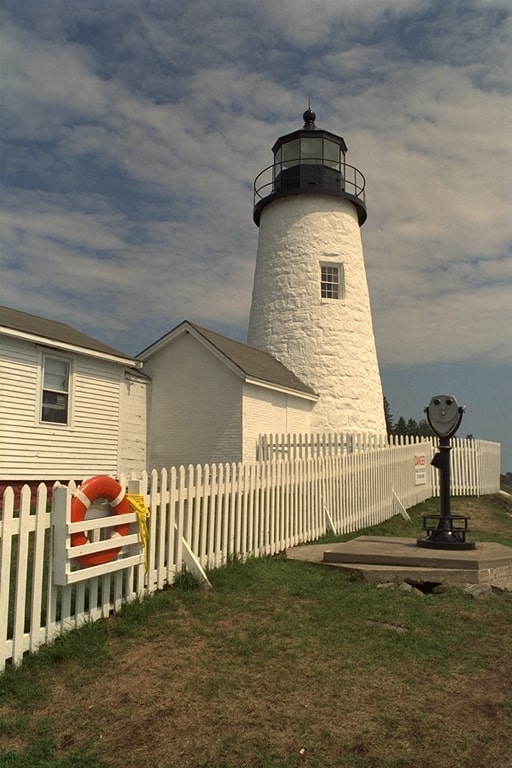

Supplement: Supplemental Information 2 — For Training purposes- Berkeley Segmentation dataset300 was used named soniya-mask. CBSD68, Set12, McMaster, and Kodak24 were used for testing purposes. [file peerj-cs-11-2449-s002.zip › overall database/kodak24/kodim19.jpg]

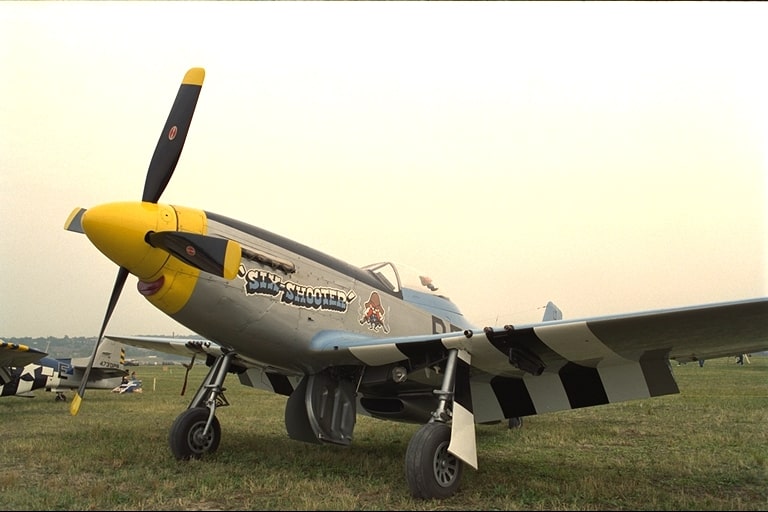

Supplement: Supplemental Information 2 — For Training purposes- Berkeley Segmentation dataset300 was used named soniya-mask. CBSD68, Set12, McMaster, and Kodak24 were used for testing purposes. [file peerj-cs-11-2449-s002.zip › overall database/kodak24/kodim20.jpg]

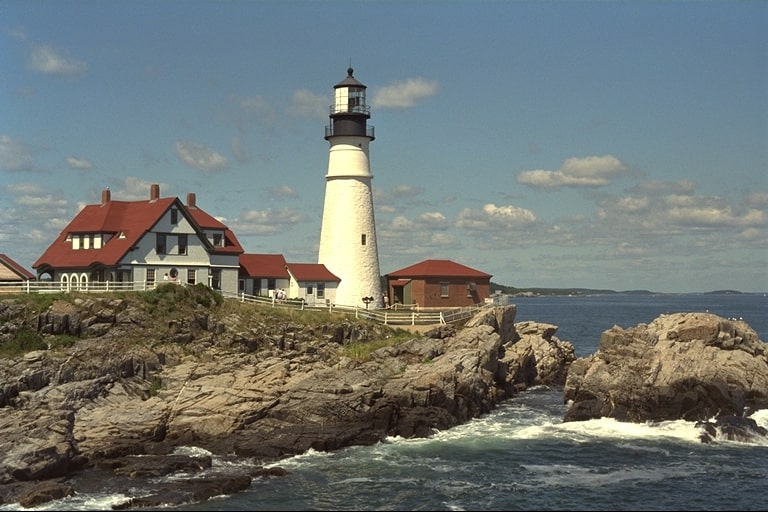

Supplement: Supplemental Information 2 — For Training purposes- Berkeley Segmentation dataset300 was used named soniya-mask. CBSD68, Set12, McMaster, and Kodak24 were used for testing purposes. [file peerj-cs-11-2449-s002.zip › overall database/kodak24/kodim21.jpg]

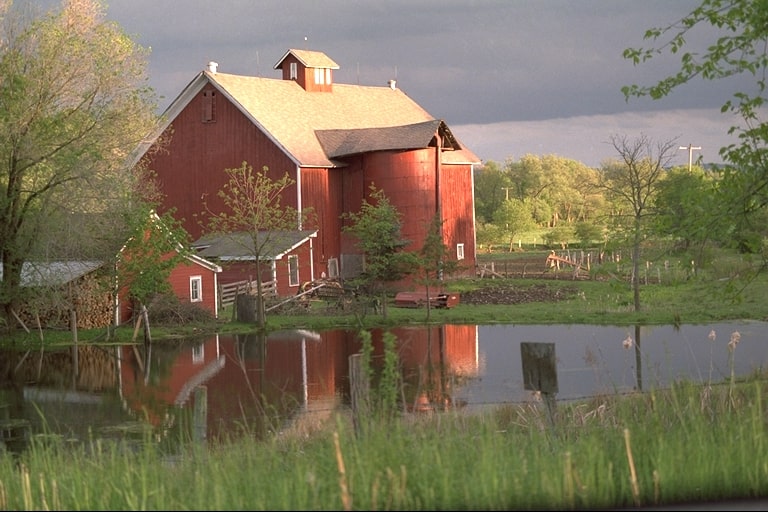

Supplement: Supplemental Information 2 — For Training purposes- Berkeley Segmentation dataset300 was used named soniya-mask. CBSD68, Set12, McMaster, and Kodak24 were used for testing purposes. [file peerj-cs-11-2449-s002.zip › overall database/kodak24/kodim22.jpg]

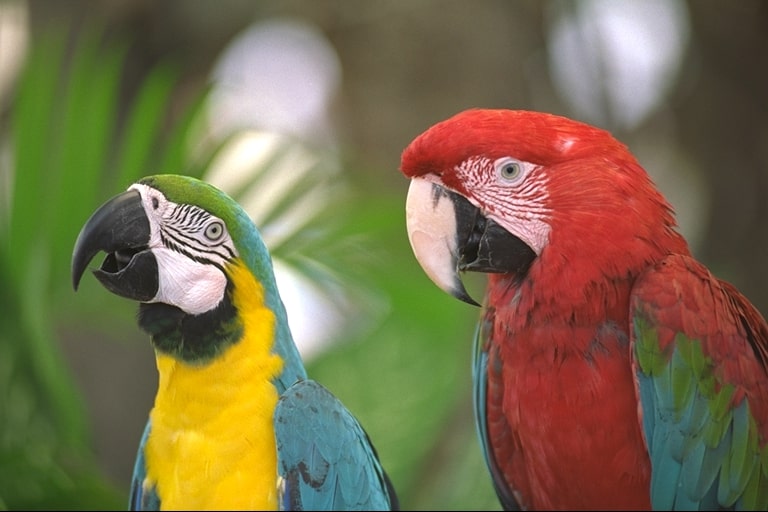

Supplement: Supplemental Information 2 — For Training purposes- Berkeley Segmentation dataset300 was used named soniya-mask. CBSD68, Set12, McMaster, and Kodak24 were used for testing purposes. [file peerj-cs-11-2449-s002.zip › overall database/kodak24/kodim23.jpg]

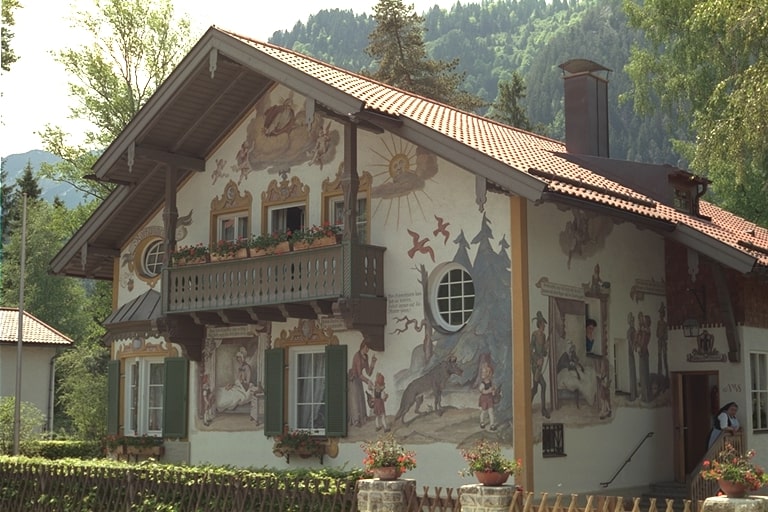

Supplement: Supplemental Information 2 — For Training purposes- Berkeley Segmentation dataset300 was used named soniya-mask. CBSD68, Set12, McMaster, and Kodak24 were used for testing purposes. [file peerj-cs-11-2449-s002.zip › overall database/kodak24/kodim24.jpg]

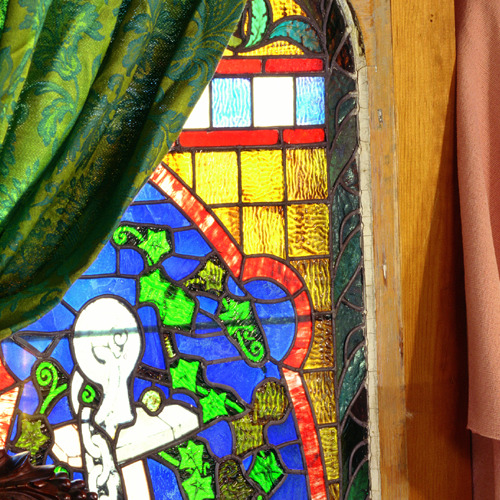

Supplement: Supplemental Information 2 — For Training purposes- Berkeley Segmentation dataset300 was used named soniya-mask. CBSD68, Set12, McMaster, and Kodak24 were used for testing purposes. [file peerj-cs-11-2449-s002.zip › overall database/Mcmaster/1.jpg]

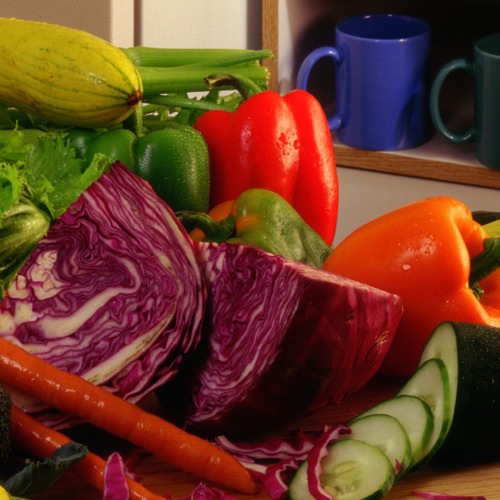

Supplement: Supplemental Information 2 — For Training purposes- Berkeley Segmentation dataset300 was used named soniya-mask. CBSD68, Set12, McMaster, and Kodak24 were used for testing purposes. [file peerj-cs-11-2449-s002.zip › overall database/Mcmaster/10.jpg]

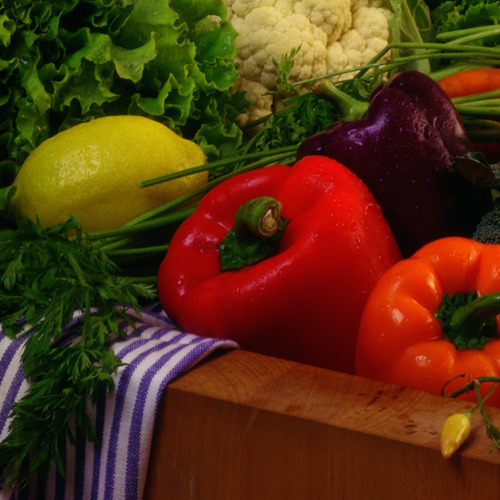

Supplement: Supplemental Information 2 — For Training purposes- Berkeley Segmentation dataset300 was used named soniya-mask. CBSD68, Set12, McMaster, and Kodak24 were used for testing purposes. [file peerj-cs-11-2449-s002.zip › overall database/Mcmaster/11.jpg]

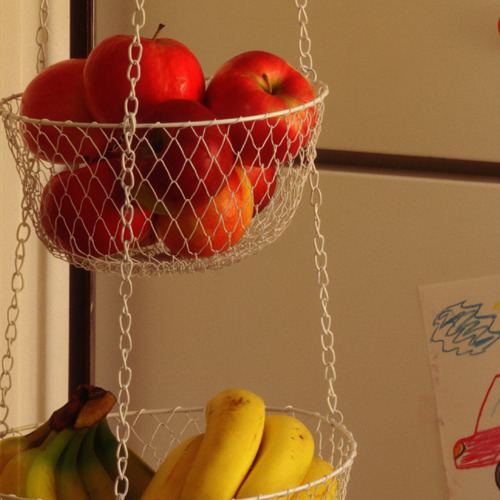

Supplement: Supplemental Information 2 — For Training purposes- Berkeley Segmentation dataset300 was used named soniya-mask. CBSD68, Set12, McMaster, and Kodak24 were used for testing purposes. [file peerj-cs-11-2449-s002.zip › overall database/Mcmaster/12.jpg]

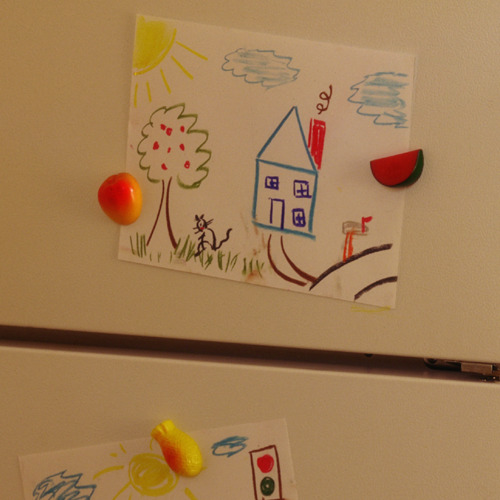

Supplement: Supplemental Information 2 — For Training purposes- Berkeley Segmentation dataset300 was used named soniya-mask. CBSD68, Set12, McMaster, and Kodak24 were used for testing purposes. [file peerj-cs-11-2449-s002.zip › overall database/Mcmaster/13.jpg]

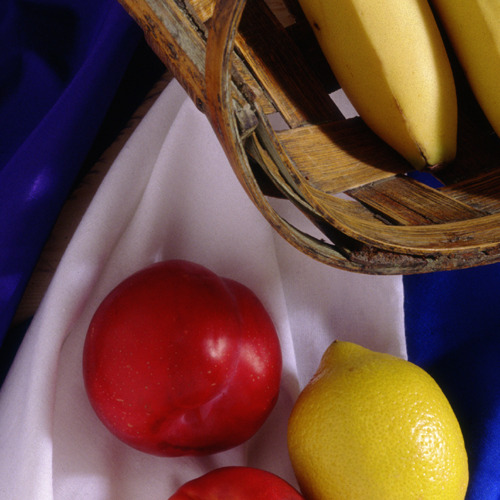

Supplement: Supplemental Information 2 — For Training purposes- Berkeley Segmentation dataset300 was used named soniya-mask. CBSD68, Set12, McMaster, and Kodak24 were used for testing purposes. [file peerj-cs-11-2449-s002.zip › overall database/Mcmaster/14.jpg]

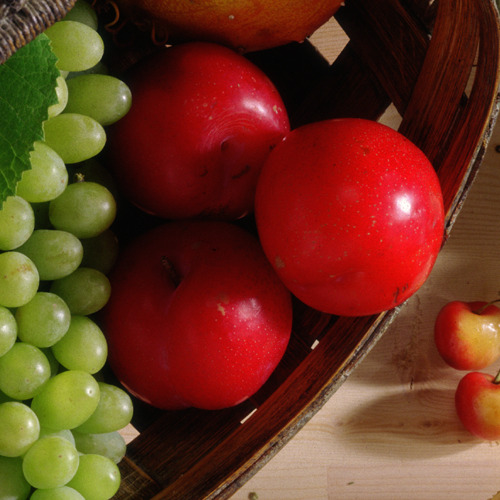

Supplement: Supplemental Information 2 — For Training purposes- Berkeley Segmentation dataset300 was used named soniya-mask. CBSD68, Set12, McMaster, and Kodak24 were used for testing purposes. [file peerj-cs-11-2449-s002.zip › overall database/Mcmaster/15.jpg]

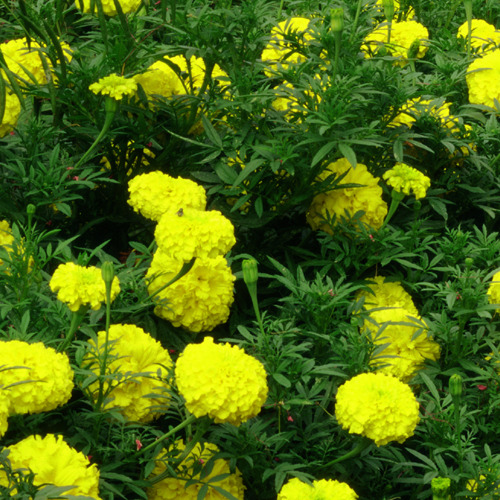

Supplement: Supplemental Information 2 — For Training purposes- Berkeley Segmentation dataset300 was used named soniya-mask. CBSD68, Set12, McMaster, and Kodak24 were used for testing purposes. [file peerj-cs-11-2449-s002.zip › overall database/Mcmaster/16.jpg]
